# The functional diversification of somatosensory neuron repertoires across Mammalia

**DOI:** 10.64898/2026.09.19.752904

**Published:** 2026-09-21

**Authors:** S. Andrew Shuster, Jia Yin Xiao, Bruno Gegenhuber, Shamsuddin A. Bhuiyan, Min Dai, Yongting Chen, Michelle M. DeLisle, Preston Sheng, Elizabeth Howell, Michael Brecht, Elena O. Gracheva, Cynthia F. Moss, David L. Paul, Ishmail Abdus-Saboor, Gord Fishell, William Renthal, Michael E. Greenberg, David D. Ginty

**Affiliations:** 1 Department of Neurobiology, Harvard Medical School; Boston, MA, USA.; 2 Howard Hughes Medical Institute, Harvard Medical School; Boston, MA, USA.; 3 Department of Neurology, Brigham and Women’s Hospital and Harvard Medical School; Boston, MA, USA.; 4 Department of Biological Sciences, Zuckerman Mind Brain Behavior Institute, Columbia University; New York, NY, USA.; 5 Bernstein Center for Computational Neuroscience Berlin, Humboldt-Universität zu Berlin, Berlin, Germany.; 6 Department of Cellular and Molecular Physiology, Department of Neuroscience, Center for Neuroethology and Physiological plasticity, Yale University School of Medicine, New Haven, CT, USA.; 7 Department of Psychological and Brain Sciences, Johns Hopkins University, Baltimore, MD, USA.

## Abstract

The striking diversity of mammalian body forms and surfaces, lifestyles, and habitats prompts the question of how each species' somatosensory neuron repertoire accommodates such diversity. The visual and olfactory systems diversify by gaining and losing sensor/receptor genes and corresponding sensory cell types. Using multiomic single-cell analysis of dorsal root ganglia neurons from thirteen different mammalian species and cross-species functional studies enabled by cell-type-specific enhancer viruses, we show that mammals instead assemble species-specific, functionally distinct somatosensory neuron repertoires from a conserved set of neuron types (orthotypes). Repertoire diversification takes multiple forms: orthotype abundance scales with variable bodily traits like hair follicle density, facilitating relevant behaviors; sensor/receptor expression is shuffled across species, such that orthotypes can detect different stimuli in different species; and orthotypes split into functionally distinct, species-specific subtypes (paratypes). Thus, unlike other sensory systems, the mammalian somatosensory system diversifies through multifarious changes to conserved orthotypes, enabling adaptation to diverse habitats, lifestyles, and body forms.

Natural selection shapes animal body structures and sensory systems alike to enhance survival and reproduction across diverse habitats. For instance, mammalian body surfaces vary widely, from the dense fur of the chinchilla, adapted to alpine Andean peaks, to the furless skin of the fossorial naked mole-rat, adapted to tunnels under the deserts and savannahs of East Africa. Many sensory systems, including the visual and olfactory systems, diversify by gaining and losing sensory receptor genes and corresponding sensory cell types which each express a single receptor (1–8). Somatosensory neurons transduce stimuli from across the body surfaces of all mammals, yet how mammalian somatosensory neuron repertoires have diversified to accommodate a wide range of environments, lifestyles, and body surfaces remains poorly understood.

Unlike other sensory systems, the mammalian somatosensory system is multimodal, coordinately detecting mechanical, thermal, and chemical stimuli, and many individual somatosensory neurons are themselves functionally polymodal, expressing multiple sensor/receptor genes simultaneously (9–12). This complexity has been dissected largely in mice: single-cell transcriptomic studies of mouse dorsal root ganglia (DRG) and trigeminal ganglion neurons (11, 13–20) and functional studies using the vast mouse genetic toolkit have identified over a dozen molecularly, anatomically, and physiologically defined cell types (11, 12, 17, 21–32). While this body of knowledge constitutes a foundation for defining mammalian somatosensory neuron types and their functions, the extent to which mouse somatosensory neuron types and their constituent features generalize across Mammalia remains unclear.

We asked how somatosensory neuron repertoires diversify across mammals by collecting and analyzing DRG neuron multiomic data from thirteen species spanning rodent, primate, and other placental clades, followed by cross-species functional studies enabled by new enhancer-based viral tools. We report that different mammals use a conserved set of neuron types (orthotypes) to assemble functionally distinct somatosensory neuron repertoires and show that these vary in three major ways: 1) orthotype abundance can scale with variable bodily traits like hair follicle density, facilitating relevant behaviors; 2) sensor/receptor expression is shuffled across species, such that orthotypes can detect different stimuli in different species; and 3) orthotypes split into functionally distinct, species-specific subtypes (paratypes). Thus, mammalian somatosensory neuron repertoires diversify not primarily by inventing new receptors and cell types, as other sensory systems do, but by recombining existing repertoires.

## Results

### Mammalian somatosensory neuron repetoires across rodent, primate, and other placental clades

To systematically describe how primary somatosensory neuron repertoires vary across Mammalia, we performed single-nucleus RNA- and ATAC-sequencing on DRGs harvested from all axial levels of nine species: four muroid rodents (mouse, rat, deer mouse, and hamster); two distantly related non-muroid rodents serving as an evolutionary outgroup (naked mole-rat and ground squirrel); and three small Laurasiatherian species (two bat species and Etruscan shrew) (Fig. 1A). We also re-analyzed publicly available DRG neuron single-cell datasets from guinea pig (33) and three old-world primates (two macaque species and human) (33–35). These 13 species belong to four clades that contain the most species-rich mammalian orders and ~80% of all mammalian species (rodents ~41%, bats ~22%, Eulipotyphla ~9%, primates ~8%) (36). They collectively span lifestyles from human commensals (mice, rats) and hibernators (hamsters, ground squirrels) to fossorial (naked mole-rats) and flying (bats) mammals; body surfaces from hairy (most species) to nearly hairless (naked mole-rats and humans); and body masses from ~2–3 g (Etruscan shrew) to 40+ kg (human).

We used a standardized computational pipeline for quality control, normalization, dimensionality reduction, and clustering of each sample from each species separately before combining datasets across replicates and species for comparative analysis. Although our datasets contain non-neuronal DRG cells, our analyses focused on DRG neuron clusters.

#### Conservation of DRG neuron types (orthotypes) across mammals

We first identified orthotypes of twelve major mouse somatosensory neuron types across species (Fig. 1 and fig. S1); ten of these twelve were conserved across all species, and only two species appeared to lack one or two orthotypes (see below). Large-diameter, heavily myelinated, fast-conducting A-fiber orthotypes included proprioceptors (A-Pro), which detect muscle, tendon, and joint movement and position (37); Aβ-Field, Aβ-, and Aδ-low-threshold mechanoreceptors (LTMRs) that detect light, innocuous mechanical forces (11, 24, 28, 31); and A-fiber high-threshold mechanoreceptors (Aδ-HTMRs) that detect noxious mechanical and thermal stimuli and function as nociceptors (11, 12, 31, 32). Small-diameter, slow-conducting C-fiber orthotypes included cold thermoreceptors (C-Cold) (11, 12); nonpeptidergic putative warm (C-Heat (Calca–)) and peptidergic heat (C-Heat (Calca+)) (11, 12) thermoreceptors; C-fiber LTMRs (C-LTMRs) (24); and three types of jointly mechano- and heat-sensitive (“nonpeptidergic”) polymodal C-fiber neurons (C-M/H neurons), which we distinguish here based on their mouse marker genes (*Mrgprd*, *Mrgpra3*, and *Sst*) (11, 12, 25, 26, 38).

Distinct analytical approaches relying either on conserved marker genes or on large numbers of highly variable genes all indicated widespread conservation of these orthotypes across most species. Cell types co-clustered across biological replicate samples from individuals of the same species (fig. S2, A to F), indicating high, consistent data quality. Combining samples from different species yielded graphs in which conserved cell types (orthotypes) co-clustered across species, suggesting conserved orthotypes across species and clades (Fig. 1, B and C). Many genes that mark specific DRG neuron types in mice had conserved orthotype-specific expression patterns across species (Fig. 1D) (13, 17, 33–35, 39, 40). In accordance with cell-type conservation, the expression patterns of several cell-type-restricted transcription factors (17) were largely conserved across orthotypes, as were those of growth factor receptors required for somatosensory neuron survival, growth, and cell-type identity (fig. S2, G and H) (41, 42). Analysis of our snATAC-seq datasets likewise indicated conservation at the level of chromatin accessibility: orthotype-specific transcription factor binding-motif enrichment was largely consistent across species, suggesting that orthotypes use similar sets of transcription factors to establish and maintain core, conserved gene expression programs, and independent clustering of cells based on ATAC data recapitulated the neuronal clusters identified by transcriptomics (fig. S2, I to K). These transcriptomic and genomic data provide evidence of somatosensory neuron-type conservation across Mammalia.

Together, our single-nucleus RNA- and ATAC-sequencing datasets indicate that most of the DRG neuron types described and characterized in mice are broadly conserved across mammals. The near-complete conservation of somatosensory neuron orthotypes across a wide range of mammals suggests that each orthotype confers an adaptive advantage that has been maintained by selection. We provide an accompanying website (link) enabling conversion between different somatosensory neuron nomenclatures for easy access to these single-cell datasets as a community resource.

### Wide variation in DRG neuron orthotype proportions across mammals

Despite the remarkable conservation of somatosensory neuron orthotypes across most mammals, we observed marked variation in the proportions of several orthotypes across species (Fig. 2A and fig. S3). In a few extreme cases, an orthotype appeared to be absent in certain species. Most notably, C-LTMRs, which comprise ~20% of all mouse DRG neurons, were absent in naked mole-rats and rare in humans (Fig. 2A and fig. S3). C-LTMRs are a large population of exquisitely mechanosensitive C-fiber neurons that respond to gentle stroking and cooling of the skin in mice (24) and have been implicated in pleasant/affective social touch (43, 44), tissue repair (45), tactile allodynia (46, 47), analgesia (43), pain recovery (48), anxiolysis (46), tickle (49), and wet-dog shake behavior (50). Moreover, studies of human C-LTMRs are inconsistent, with some assays describing sizeable C-LTMR populations (51) and others noting their rarity or absence (35, 51, 52). Given this apparent variability, we focused on characterizing cross-species differences in the proportions of this orthotype.

#### Scaling of C-LTMR proportions and hair follicle density across mammals

C-LTMR proportions varied widely across species in our single-cell datasets: mice had the highest proportion (~20% of all DRG neurons); most small mammals (rodents, bats, and shrews) had 9–16%; macaques (~7%) and humans (~1%) had fewer; and naked mole-rat samples lacked C-LTMR clusters altogether (Fig. 2A and figs. S3 and S4, A to C). Notably, C-LTMRs are the only C-fiber neurons shown to innervate hair follicles, establishing lanceolate endings around the fine (zigzag and awl/auchene) hair follicles that comprise the downy undercoat and ~99% of mouse trunk-skin hair follicles (24). C-LTMRs are also present at different proportions across axial levels in mice, with more at non-limb levels innervating hairy skin and fewer at limb-projecting levels (17, 24). Given this intriguing relationship between hair follicle density and C-LTMR abundance in mice, we asked whether C-LTMR proportions also scale with hair follicle density across mammals, which exhibit several orders of magnitude in variation of this trait (53).

We performed *in situ* hybridization (ISH) against two conserved C-LTMR co-marker genes to quantify C-LTMR proportions in mid-thoracic DRGs, which innervate trunk hairy skin, across a range of mammals (mice, rats, naked mole-rats, macaques, and humans) with known hair follicle densities (Fig. 2, B to G and fig. S4, D and E) (53, 54). Consistent with our single-cell data and previous studies (17, 24), mouse mid-thoracic DRGs had abundant C-LTMRs (~20%), proportionally more than those of rats (~13%), which have a lower hair follicle density. By contrast, mid-thoracic DRGs of the naked mole-rat, a fossorial rodent lacking a downy undercoat (54), had exceedingly few (~1%) putative C-LTMRs (Fig. 2D and fig. S4F). Assessment in DRGs of relatively hairy (rhesus macaque) and less hairy (human) primates (53, 55) revealed far more C-LTMRs in macaques (~7%) than in humans (~2%) (Fig. 2, E to G). Thus, these data demonstrate that the relative abundance of an orthotype (C-LTMR) may scale with a salient, rapidly evolving mammalian body surface feature (hair follicle density).

#### A C-LTMR-driven behavior conserved across hairy rodents

Motivated by the wide variation in C-LTMR proportions, we next asked what ethological functions C-LTMRs may play across mammals. C-LTMR functions have been assessed in mice largely by testing for deficits in candidate behaviors following C-LTMR genetic ablation or inhibition, and in humans by inferring cell-type identity from electrophysiological responses in peripheral microneurography studies while recording participants' descriptions of evoked sensations (46, 56–58). We pursued a distinct gain-of-function approach, focusing on behavior resulting from stimulation of genetically identified C-LTMRs. C-LTMR stimulation in mice was recently reported to trigger wet-dog shake (WDS) behavior (50), a motor sequence in which animals repeatedly twist the head, neck, and upper trunk to forcefully expel water or other materials from the hair on their dorsal body surface (59). We therefore asked whether C-LTMRs innervate hair follicles and evoke WDS beyond mice, and whether naturalistic stimuli can evoke WDS in species lacking C-LTMRs.

Studying C-LTMR form and function beyond mice, wherein genetic tools are readily available, required new approaches. We therefore mined our single-nucleus multiome datasets to nominate ATAC peaks enriched in C-LTMRs as candidate enhancers and generated enhancer AAVs (eAAVs) (60–65) to test whether they could drive cell-type-specific payload expression across species (Fig. 3, A and B, and fig. S5A). Screening seven candidate elements revealed several that drove payload expression in mouse C-LTMRs with high cell-type-specificity (>75%; Fig. 3, C and D), including one located in an intron of a gene expressed specifically in rodent C-LTMRs (Fig. 3A and fig. S5, B and C). Importantly, this element also drove high, cell-type-specific reporter expression in rat C-LTMRs (Fig. 3, E and F), enabling cross-species comparisons of C-LTMR properties and functions.

Using this eAAV, we first found that genetically labeled rat C-LTMRs, like those of mice, form hair follicle lanceolate endings and innervate spinal cord lamina II (fig. 6), indicating conserved peripheral morphology and central connectivity across these two species (11, 24). We then asked if C-LTMR activation triggers WDS beyond mice. Optogenetic stimulation of the upper back in non-transgenic, virally injected mice and rats expressing an optogenetic activator targeted to C-LTMRs elicited WDS in experimental but not control animals (Fig. 3, G and H, Movies S1 and S2), revealing a conserved behavioral function across species (50). Further testing showed that rats, like mice, perform WDS following water or oil application to upper-back hairy skin (Fig. 3, I and J and fig. S7A). Because mice with C-LTMRs ablated show WDS deficits (50), we then asked whether species lacking C-LTMRs exhibit WDS in response to such stimuli. Strikingly, both water and oil applied to the upper back of naked mole-rats—which have few, if any, C-LTMRs and a fossorial lifestyle in which they rarely, if ever, get wet but constantly encounter debris on their dorsal skin (72, 73)—failed to elicit WDS (Fig. 3, I and J and fig. S7B). Thus, furry C-LTMR-rich species (mice, rats, and macaques (68)) perform WDS, whereas species with sparse fur and few C-LTMRs (naked mole-rats and humans) appear not to (59). This is consistent with the proposal that WDS evolved to clear water trapped against the skin by fur (59), a need obviated in sparsely-haired mammals. Thus, the remarkable divergence of C-LTMR proportions across mammals corresponds to both hair follicle density and behavioral responses to hair-associated mechanical stimuli.

Together, our transcriptomic, anatomical, and behavioral analyses illustrate how diverse mammals employ conserved DRG neuron orthotypes in species-specific proportions adapted to their body surfaces, lifestyles, and habitats.

### Widespread sensor/receptor shuffling within orthotypes across species

While somatosensory neuron orthotypes can vary in abundance across species, they may also diverge in function if genes critical for sensory responses diverge in expression. We therefore asked whether DRG neuron orthotypes of different species across our datasets differ in the expression of ion channel and sensor/receptor genes. The expression of several ion channels that dictate cell-type intrinsic physiological properties (69) was largely conserved across species (fig. S8A). However, surprisingly, orthotype-specific expression of several critical sensor genes—including the mechanosensor *Piezo2* (70, 71) and the thermo-/chemo-sensors *Trpa1*, *Trpm8*, and *Trpv1* (72)—diverged starkly across species (Fig. 4A), with numerous differences even between closely related species such as mice and rats. For example, mouse polymodal C-M/H (Mrgprd) neurons highly expressed *Trpa1* but not *Trpv1*, whereas the rat orthotype had the reciprocal pattern (Fig. 4A, rightmost green box). Many such differences were observed, especially within the three polymodal C-fiber (C-M/H) orthotypes. The Aδ-HTMR nociceptor orthotype also varied in *Trpm8* and *Trpa1* expression across species, suggesting potential species-specific mechanisms for detecting noxious cold and electrophilic substances. We call this phenomenon *sensor shuffling* and validated cases of it across several sensor genes (*Piezo2*, *Trpa1*, and *Trpv1*) in several mouse, rat, and human orthotypes by ISH (Fig. 4, A to J, and fig. S8, B to P).

In some cases, orthotype-specific sensor expression patterns in mice (e.g., *Trpa1* and *Trpv1* in C-M/H (Mrgprd) neurons, *Trpm8* in Aδ-HTMRs, *Piezo2* in C-M/H (Mrgpra3)/C-M/H (Sst) neurons) more closely resembled those of deer mice or hamsters than of rats, the closest relative of mice in our datasets, ruling out phylogenetically constrained regulation of sensor expression. As these data indicate remarkable dynamicity in expression of these critical functional genes within and across orthotypes, even among muroid rodents, we asked how sensor shuffling between and across orthotypes emerges. Analysis of our snATAC-seq data suggested that, while cell-type-specific regulation of sensor expression by particular *cis*-regulatory elements can be conserved across species, orthotypes may also use distinct sets of *cis*-regulatory elements to regulate sensor expression (fig. S9), implicating divergent, species-specific distal *cis*-regulatory element (putative enhancer) utilization in the establishment of sensor shuffling.

This degree of sensor shuffling is unusual. Across many vertebrate sensory systems, notably vision and olfaction, sensory cells largely follow a one-receptor-one-cell-type rule (2), in which expression of a given sensor/receptor gene defines a cell type across species, and widespread inter-orthotype sensor/receptor gene shuffling has not been reported for other sensory modalities with conserved receptor genes (e.g., vision, taste). Somatosensory neurons appear to break this rule with their widespread sensor shuffling. Indeed, our findings suggest that sensor shuffling may endow orthotypes with flexibility of function across species.

This remarkable dynamism extends beyond canonical sensory receptors to receptors for internal, intercellular signals—notably those from immune cells, which DRG neurons detect alongside external stimuli. In fact, expression of immune/cytokine receptor genes was strikingly divergent across species (Fig. 4K). For example, *Cysltr2*, a receptor for immune cell-derived lipids (73–75), is a specific marker of mouse C-M/H (Sst) neurons (11) but was expressed in several other orthotypes across rats, ground squirrels, primates, bats, and shrews (Fig. 4K and fig. S10). Several other conserved immune/cytokine receptor genes (*Hrh1*, *Il31ra*, *Osmr*) likewise exhibited shuffled expression—including *Hrh1*, the histamine receptor implicated in histamine-mediated itch in humans (76, 77), which our data show is expressed in different combinations of polymodal C-M/H orthotypes in mice, rats, and humans—suggesting that the corresponding immune signals are detected by different C-M/H orthotypes in different mammals. Notably, whereas immune receptor expression was largely restricted to the three polymodal C-M/H orthotypes in most rodents, some non-rodent C-LTMRs also expressed these receptors (*Cysltr2* in humans and fruit bats, *Hrh1* in shrew), suggesting that the ancestral genetic program specifying C-LTMRs retains the potential to confer immune signal detection, indicating a broader functional potential for C-LTMRs than previously appreciated.

The polymodal C-M/H orthotypes stood out in these analyses, exhibiting the most frequent shuffling of both sensors and receptors, while many other orthotypes showed largely invariant sensor/receptor expression. To ask whether this dynamicity reflects broader transcriptome-wide divergence rather than divergence of just a handful of genes, we built transcriptome distance trees across species and summed total branch length per orthotype (fig. S11) (78). A-fiber orthotypes showed, on average, lower transcriptomic divergence than C-fiber orthotypes, and the three polymodal C-M/H orthotypes were the most divergent overall, as they were with sensor/receptor expression. Together, these analyses suggest that the polymodal C-M/H orthotypes, which arise from a shared developmental lineage (41, 42), are especially evolutionarily dynamic, both in critical sensor/receptor genes and at the whole-transcriptome level. Their extensive sensor/receptor shuffling also suggests that these orthotypes could play functionally distinct roles across species.

#### Functional consequences of sensor shuffling across mouse and rat C-M/H (Mrgprd) neurons

Thus, to determine whether orthotypes with divergent sensor/receptor expression indeed have divergent sensory capabilities, we compared the physiological response properties of mouse and rat C-M/H (Mrgprd) neurons. Our transcriptomic and histological data indicated that mouse neurons highly express *Trpa1* but not *Trpv1*, while rat neurons highly express *Trpv1* but not *Trpa1*, predicting that mouse but not rat neurons would respond to the TRPA1 agonist allyl isothiocyanate (AITC) (79, 80), whereas rat neurons would respond to the TRPV1 agonist capsaicin (81). Utilizing our multiome ATAC data, we generated eAAVs providing ≥85% (up to ~97%) cell-type-specific access to C-M/H (Mrgprd) neurons in mice and rats (Fig. 5, A to E and S12, A to E), again demonstrating the cross-species utility of our eAAV platform. Labeled neurons of both species innervated spinal cord dorsal horn lamina II, indicating conserved central anatomy of this orthotype (fig. S13, A and B).

We used a C-M/H (Mrgprd)-targeting eAAV to express the tetracycline transactivator to drive TRE-amplified GCaMP8s in mouse and rat C-M/H (Mrgprd) neurons and performed *in vivo* Ca^2+^ imaging of lumbar level 4 neurons to assess their physiological responses to mechanical and chemical stimulation (Fig. 5, F to K, and fig. S12, F to I). As predicted, C-M/H (Mrgprd) neurons of both species responded robustly to hindlimb mechanical stimulation. Remarkably, mouse neurons responded robustly to AITC but not capsaicin, whereas rat neurons responded robustly to capsaicin, with only a minor subpopulation responding to AITC (Fig. 5, H to K, and S13, C and D). These results establish that a conserved orthotype has been functionally repurposed across species through sensor/receptor shuffling and showcase that comparative transcriptomics can predict and cross-species cell-type-specific physiology enabled by eAAVs can reveal species-specific sensory functions at the level of individual orthotypes. More generally, these findings validate our single-cell transcriptomic results suggesting that sensor/receptor shuffling endows somatosensory neuron orthotypes with flexibility of function across species.

### Diversification of polymodal C-fiber orthotypes into species-specific paratypes

The minor AITC-responsive subpopulation of rat C-M/H (Mrgprd) neurons (Fig. 5, I and K, and fig. S13D) violated predictions based on the minimal *Trpa1* expression in the pseudobulk rat C-M/H (Mrgprd) dataset (Fig. 5F), despite cell-type-specific eAAV payload expression (fig. S12, H and I). Thus, we asked whether this AITC-responsive subpopulation corresponds to a species-specific C-M/H (Mrgprd) subtype—a paratype, in analogy to a gene paralog—that expresses *Trpa1*, which would indicate cross-species diversification of DRG neuron repertoires by species-specific orthotype splitting.

#### Molecularly, anatomically, and physiologically distinct rat C-M/H (Mrgprd) neuron paratypes

Rat C-M/H (Mrgprd) neurons comprised two distinct clusters that we did not readily find in other species (Fig. 6A). While both expressed canonical C-M/H (Mrgprd) marker genes, the smaller cluster expressed *Trpa1* and other marker genes that the larger cluster did not (Fig. 6B), explaining the otherwise unexpected AITC responses, while the larger cluster expressed marker genes the smaller cluster did not. Given their shared expression of canonical orthotype markers, we interpret these not as de novo, species-specific neuron types but as candidate paratypes.

To distinguish true paratypes from mere cell states, we asked whether the clusters are anatomically distinguishable, performing ISH against orthotype and subcluster marker genes at different axial levels. One cluster was present at both limb- and non-limb-innervating levels, whereas the *Trpa1*-expressing cluster was present only at limb-innervating levels (Fig. 6, C to F, and fig. S14). Reexamination of our Ca^2+^ imaging data showed that most AITC-responsive neurons responded to⁺ hindpaw but not hairy hindlimb pinch, corroborating this anatomical distinction (Fig. 6, G to I). Together, these findings reveal diversification of rat C-M/H (Mrgprd) neurons into two molecularly, anatomically, and physiologically distinct paratypes and suggest that species-specific somatosensory neuron paratypes can specialize for innervation of distinct peripheral tissues. Paratype emergence may thus endow different skin types with distinct sensory capabilities.

#### Paratype emergence across mammalian polymodal C-fiber neurons

Is such species-specific orthotype diversification into paratypes common? Our datasets revealed additional cases within the polymodal C-M/H orthotypes. Mouse C-M/H (Mrgpra3) neurons comprised two clusters not readily found in other species (figs. S15 and S16, A and B), corresponding to types annotated previously (17) and implicated in itch (25, 82) and in pleasurable stroking touch and sexual behavior (26, 83); while both expressed canonical orthotype markers, each also expressed paratype-restricted genes, including sensor/receptor genes. Datasets from distantly related non-rodent mammals also contained apparent paratypes: shrew C-M/H (Sst) neurons comprised two clusters differing in sensor and immune receptor expression (fig. S16, C and D). Together, these findings suggest that orthotypes conserved across many mammals—most frequently the polymodal C-M/H orthotypes—diverge into paratypes in a species-specific manner.

Thus, mammalian somatosensory neuron paratypes express not only shared, orthotype-defining marker genes but also collections of paratype-specific genes, including genomically conserved sensor/receptor genes that endow distinct sensory/functional capabilities. Somatosensory neuron paratypes can also be anatomically specialized, endowing different peripheral tissues with distinct sensory capabilities. Critically, these paratypes do not appear to have emerged in a sensory receptor gene paralog-linked manner, unlike in the olfactory and visual systems. This novel mode of receptor paralog-independent paratype emergence—a snapshot of orthotype diversification in action—suggests novel mechanisms of cell-type evolution for somatosensory neurons.

## Discussion

How has the mammalian somatosensory system changed with the radiation of mammals into diverse environments, lifestyles, and body forms and surfaces? Combining DRG neuron single-cell transcriptome and chromatin accessibility datasets from thirteen mammals with new enhancer-based viral tools for cross-species genetic access and functional analyses, we find that diverse mammals assemble distinct functional repertoires from a conserved set of orthologous somatosensory neuron types (orthotypes) that diversify in three ways: orthotype abundance, sensor/receptor expression patterns, and splitting into species-specific subtypes (paratypes). Together, these findings reveal that mammalian somatosensory neuron repertoires diversify across species not primarily by creating new cell types and sensor/receptor genes but by recombining existing cell types and sensor/receptor genes to meet species-specific needs.

### Conservation of mammalian somatosensory neuron orthotypes and tools for functional studies across species

Our transcriptomic and chromatin data indicate that the mammals profiled here assemble their somatosensory neuron repertoires from a conserved set of neuron orthotypes (Fig. 1 and fig. S2). This broad conservation distinguishes somatosensory neuron repertoires from those of other sensory systems, which diverge via genomic gain/loss of sensor/receptor genes and corresponding cell types (2–7). However, conservation of transcriptomically-defined cell types does not guarantee corresponding conservation of sensory function. Thus, leveraging our snATAC-seq data, we generated cell-type-specific eAAVs for cross-species genetic access enabling characterization of orthotype anatomy, physiology, and function. Because these tools readily permit payload customization and may be used in both transgenic and non-transgenic animals, they are useful both in mice and other species in which they have been validated. Furthermore, our snATAC-seq data and methods will enable development of a fuller viral toolkit for genetic access to mammalian somatosensory neuron orthotypes, including the orthotypes that initiate pain and itch signaling. Future comparative studies of cell-type diversity can follow the template demonstrated here, incorporating functional studies to test predictions generated by observational molecular/-omics datasets.

### Species-specific orthotype proportions

Although mammalian somatosensory neuron repertoires comprise largely conserved orthotypes, their proportions can vary greatly (Fig. 2 and fig. S3). Variation in sensory orthotype proportions has been noted in the retina (8, 84–86), suggesting that cross-species variation in orthotype abundance may be a common evolutionary-developmental mechanism for tuning sensory systems to species-specific niches. The relationship we observe between C-LTMR proportions and hair follicle density suggests not only an intimate link between bodily surface features and somatosensory neuron development/specification, but also that hair-movement-associated mechanosensation may be a conserved C-LTMR function across mammals, complementing the diverse functions—notably affective touch (46, 87)—previously proposed for this type. The sparsity of C-LTMRs at limb-projecting axial levels in mice (17, 24), together with the relative rarity of this orthotype in human DRGs may also explain why single-cell RNA-sequencing studies of human DRGs, which often sample limb-innervating ganglia, variously report few or no C-LTMRs (33, 35, 39, 51, 52).

Differences in orthotype proportions appear in turn to constrain or enable particular behaviors, as we demonstrate for the C-LTMR-driven wet-dog shake (WDS): rodents with abundant C-LTMRs perform WDS, whereas naked mole-rats, which lack C-LTMRs, do not. Hair follicle density evolves rapidly in mammals (61, 93–97) and may generally decrease with body size (53). Habitat, lifestyle, and thermoregulatory strategy could therefore jointly shape hair density, C-LTMR abundance, and the adaptive value of related behaviors like the WDS, which clears the dorsal body surface of liquids and irritants. An organism's niche may thus shape the somatosensory neuron repertoire that organism assembles to meet the demands of its habitat, lifestyle, and body form and surface.

### Sensor/receptor shuffling in polymodal C-fiber (C-M/H) neurons

Because somatosensory neurons require sensor/receptor expression for sensory function, the widespread cross-species differences in their expression that we observe within orthotypes—sensor/receptor shuffling—imply divergence of orthotype function across species (Fig. 4), which our eAAV-enabled *in vivo* recordings confirmed for C-M/H (Mrgprd) neurons (Fig. 5). Sensor/receptor shuffling across species was most widespread within the three polymodal C-M/H orthotypes, which share central and peripheral anatomical (11), physiological (11, 12), and behavioral (13, 25, 38, 82, 93) properties in mice. These orthotypes also express a range of immune receptors (13) and the evolutionarily labile Mrgpr chemoreceptor family (94) that detects both immune signals and environmental irritants (95, 96). These commonalities suggest that these orthotypes may function redundantly and/or cooperatively, raising the question of why multiple such orthotypes are maintained. Because the functions of ancient multifunctional cell types tend to segregate among descendant sister types over evolution (97), it is tempting to speculate that the polymodal C-M/H orthotypes are more evolutionarily derived within mammals than other orthotypes, a possibility that could be tested by profiling distantly related species. Moreover, because immune-response genes are among the most rapidly evolving genes in mammals (98), the immune receptor shuffling largely restricted to these orthotypes suggests they may be rapidly modified across evolution and collectively tuned to detect diverse irritants and immune signals. Future testing of the functions of C-M/H orthotypes across species, perhaps using eAAVs, could also address such possibilities.

### Paratype emergence within polymodal C-fiber (C-M/H) orthotypes

We report that conserved orthotypes themselves diversify functionally by splitting into species-specific paratypes with distinct sensory capabilities, again most readily among the polymodal C-M/H orthotypes (Fig. 6 and figs. S14 to S16). While this cell-type diversification via paratype emergence is analogous to gene diversification by duplication-and-divergence, it does not appear to involve receptor paralog diversification, a critical feature of cell-type diversification in the olfactory (1–4) and visual (5–7) systems. Furthermore, partial functional redundancy among the polymodal C-M/H orthotypes may provide a permissive setting for paratype emergence, much as genetic redundancy permits paralog emergence following gene duplication. Moreover, the innervation of distinct peripheral tissues by functionally dissociable rat C-M/H (Mrgprd) paratypes suggests a link between peripheral targets and paratype development and thus a clue as to how somatosensory neuron paratypes could emerge in a receptor paralog-independent manner.

### Mechanisms of somatosensory neuron repertoire diversity across and within mammals

How the three forms of mammalian somatosensory neuron repertoire diversification identified here arise developmentally—whether specified intrinsically within post-mitotic lineages or instructed extrinsically by peripheral tissues—remains unaddressed; either mechanism could influence orthotype abundance through differential specification or survival. Axial-level (17, 24, 35) and dermatome-specific (99) (Fig. 6) patterning hints at an appreciable role for extrinsic instruction via retrograde peripheral signals. Intriguingly, the orthotypes with the greatest differences in proportions, sensor/receptor shuffling, and the most transcriptomic divergence across species (C-LTMRs and the three polymodal C-fiber orthotypes) all derive from a shared Runx1-dependent developmental lineage (100–107). As genes governing skin development and immune responses evolve rapidly (98), peripheral-tissue-directed programming of somatosensory neuron development could allow evolving skin and immune systems to flexibly tune corresponding somatosensory neuron repertoires during organismal development.

### Final Summary

In summary, mammalian somatosensory neuron repertoires assemble from a limited set of conserved orthotypes and diversify across species by three mechanisms—differences in orthotype abundance, sensor/receptor shuffling, and paratype emergence—each occurring both across species and across axial levels within species, and each most pronounced in orthotypes of a shared developmental lineage. By diversifying through such mechanisms, somatosensory neuron repertoires diverge across species in a way that other sensory systems do not, and in a manner that may be especially responsive to changes in peripheral tissues innervated by these neurons. Future work should address the developmental and evolutionary mechanisms generating these differences across Mammalia and within individual species.

## Supplementary Material

Supplement 1

## Figures and Tables

**Fig. 1.**
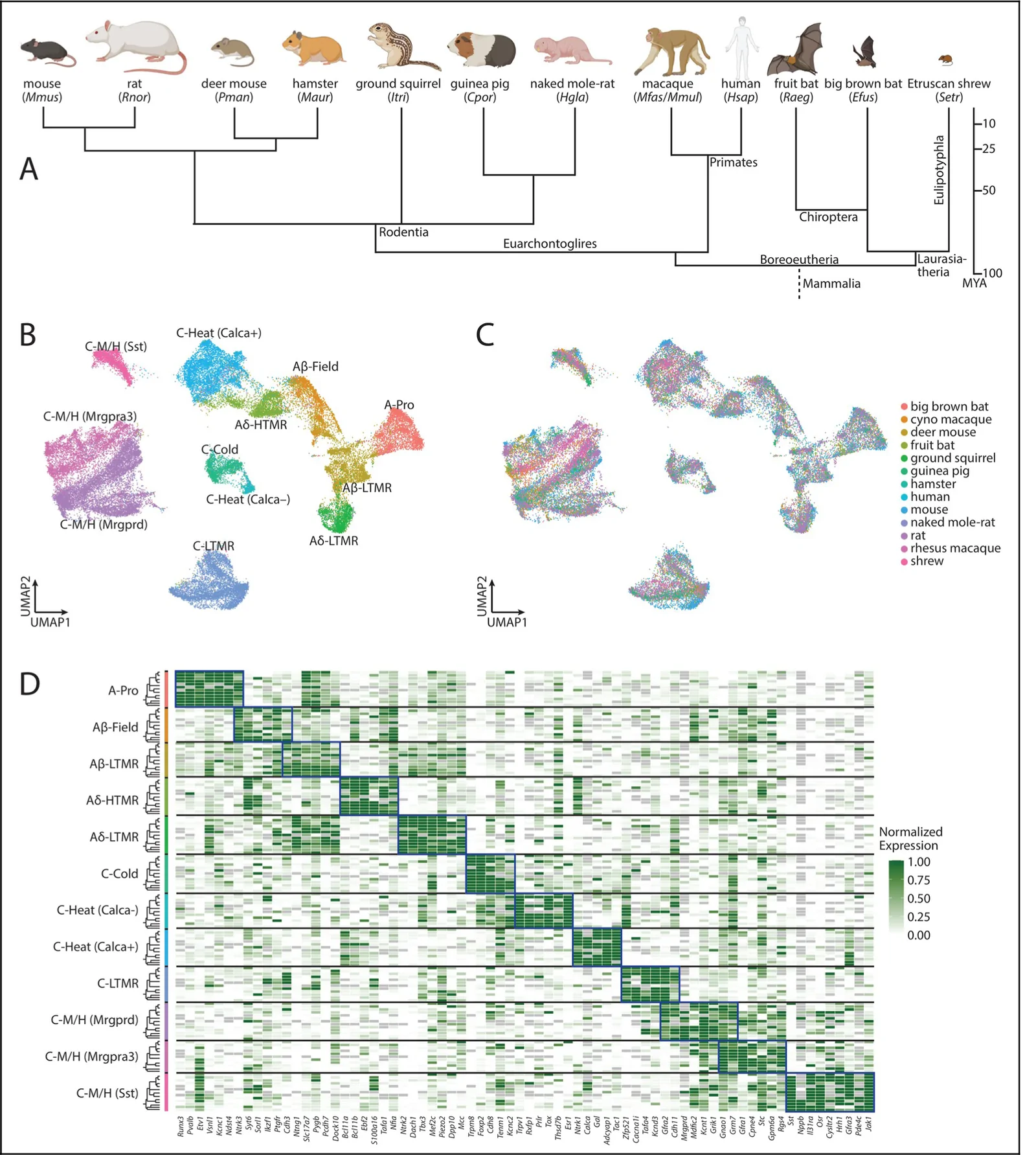
Mammalian dorsal root ganglia sensory neuron repertoires. (**A**) Phylogeny of mammalian species sampled. MYA, million years ago. (**B** and **C**) Uniform manifold approximation and projection (UMAP) embedding of single-nucleus RNA-sequencing integrated cross-species data. Colors indicate somatosensory neuron orthotype identities (B) or species-of-origin (C). (**D**) Heatmap showing average expression of marker genes (columns) within each orthotype across species (rows). Rows (left) are grouped by orthotype. Within each orthotype, species are ordered as in Figure 1A. Gray tiles indicate gene orthologs not present in any species annotation.

**Fig. 2.**
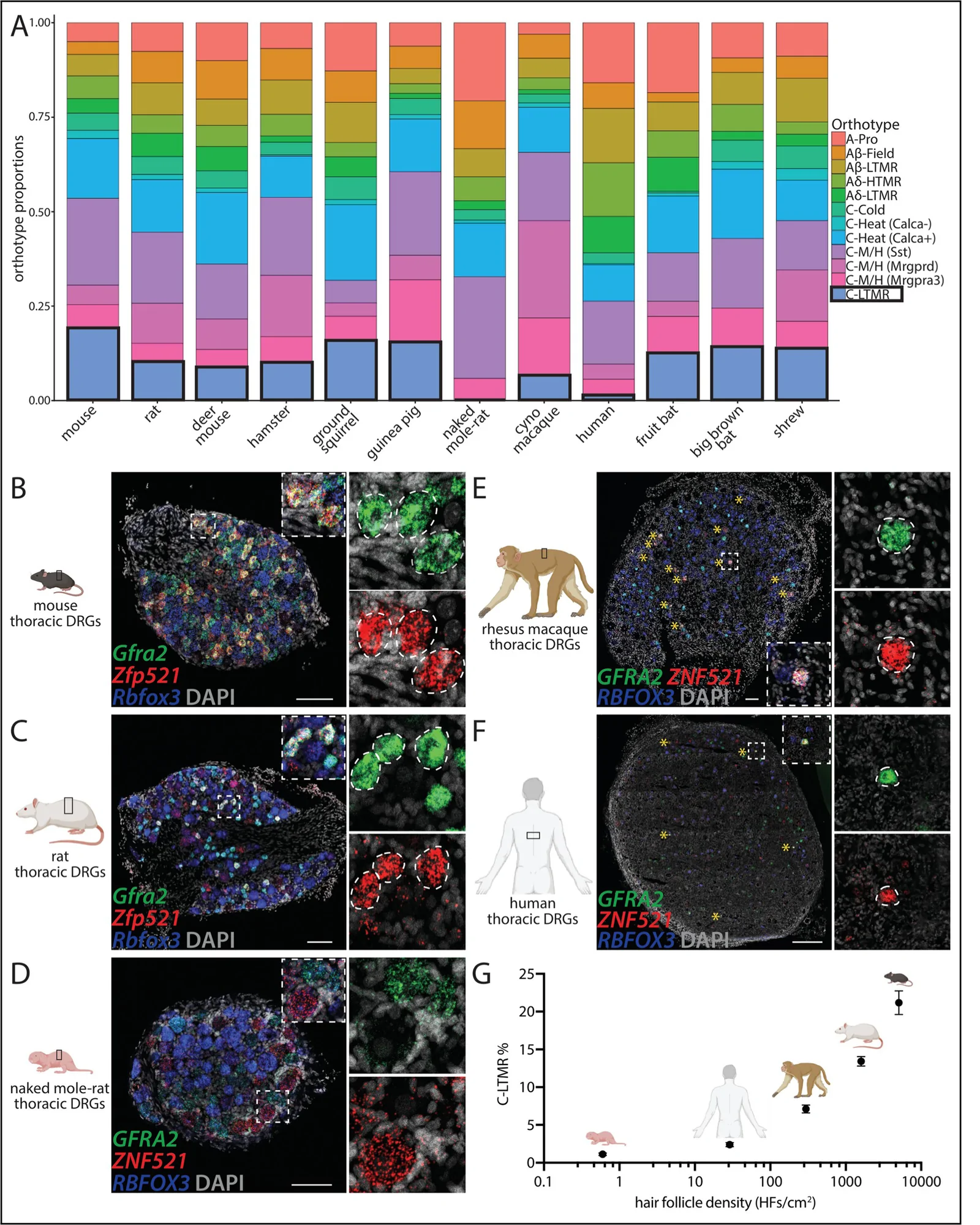
Variation across species in C-LTMR proportions in accordance with hair follicle density. (**A**) Averaged proportions of somatosensory neuron orthotypes across species. C-LTMR proportions are highlighted in black boxes (see also fig. S3). (**B** to **F**) Representative images of *in situ* hybridization against *Gfra2*/*GFRA2* (green) and *Zfp521*/*ZNF521* (red), co-markers of C-LTMRs, in mouse (B), rat (C), naked mole-rat (D), rhesus macaque (E), and human (F) mid-thoracic DRGs, along with pan-neuronal marker *Rbfox3*/*RBFOX3* (NeuN, blue). Putative C-LTMRs are outlined in dotted white lines in insets (right, all panels) and marked by yellow asterisks (E, F). Scale bars (in μm): 100 (B to E), 500 (F). (**G**) Relationship between hair follicle densities (see Methods) and C-LTMR proportions from mid-thoracic DRG sections; mean ± SEM (sections). Totals include 14–23 sections from 4–7 DRGs from 2–3 individual animals.

**Fig. 3.**
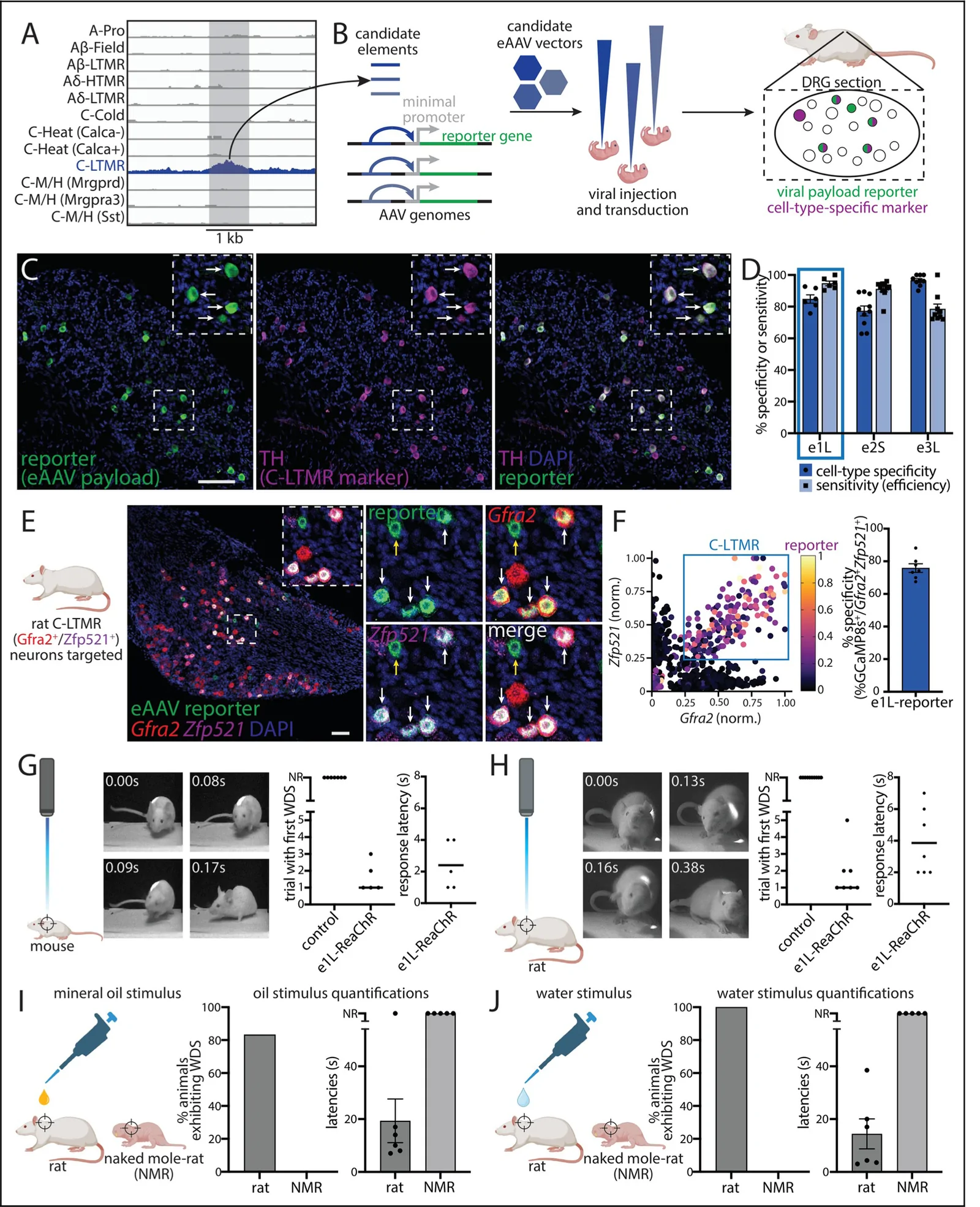
eAAV-enabled cross-species genetic access to C-LTMRs reveals control of conserved wet-dog shake behavior in hairy rodents. (**A**) Genome tracks showing a candidate cell-type-specific ATAC peak with enhanced accessibility in C-LTMR neurons (blue). (**B**) Schematic of *in vivo* eAAV screen. (**C**) Immunohistochemical validation of eAAV payload reporter expression in mouse C-LTMRs (TH^+^) (24). Insets, white arrows indicate TH^+^ neurons labeled by the eAAV reporter. Scale bar, 100 μm. (**D**) Quantification of specificity and efficiency of C-LTMR-targeting eAAVs in mice; mean ± SEM. Dotted line highlights eAAV tested and used in rats. (**E**) Joint *in situ* hybridization-immunohistochemistry validation of eAAV payload reporter expression in rat C-LTMRs (*Gfra2*^+^/*Zfp521*^+^). Insets, white arrows indicate *Gfra2*^+^/*Zfp521*^+^ neurons labeled by the eAAV reporter; yellow arrow indicates a *Gfra2*^*−*^/*Zfp521*^*−*^ off-target neuron labeled. Scale bar, 100 μm. (**F**) Quantification of specificity of C-LTMR-targeting eAAV in rats; mean ± SEM. (**G** and **H**) Left: schematics showing eAAV-mediated C-LTMR optogenetic stimulation of upper back. Middle: still frames showing mice (G) and rats (H) performing optogenetically induced wet dog shake (WDS) behavior and timecourse from WDS onset. Right: quantification of behavioral data in control (n = 7) and eAAV-C-LTMR-e1L-ReaChR (n = 5) mice and control (n = 10) and eAAV-C-LTMR-e1L-ReaChR (n = 7) rats. NR, none recorded. Bar, median trial with first WDS or mean response latency. (**I** and **J**) Left: schematics showing oil droplet (I) and water (J) application to upper back of rat and naked mole-rat (NMR) subjects. Right: quantification of behavioral data in rats (n = 6) and NMRs (n = 5). Oil droplets and water induced rat WDS behavior robustly, but never in NMRs. NR, none recorded. Latencies, mean ± SEM.

**Figure 4.**
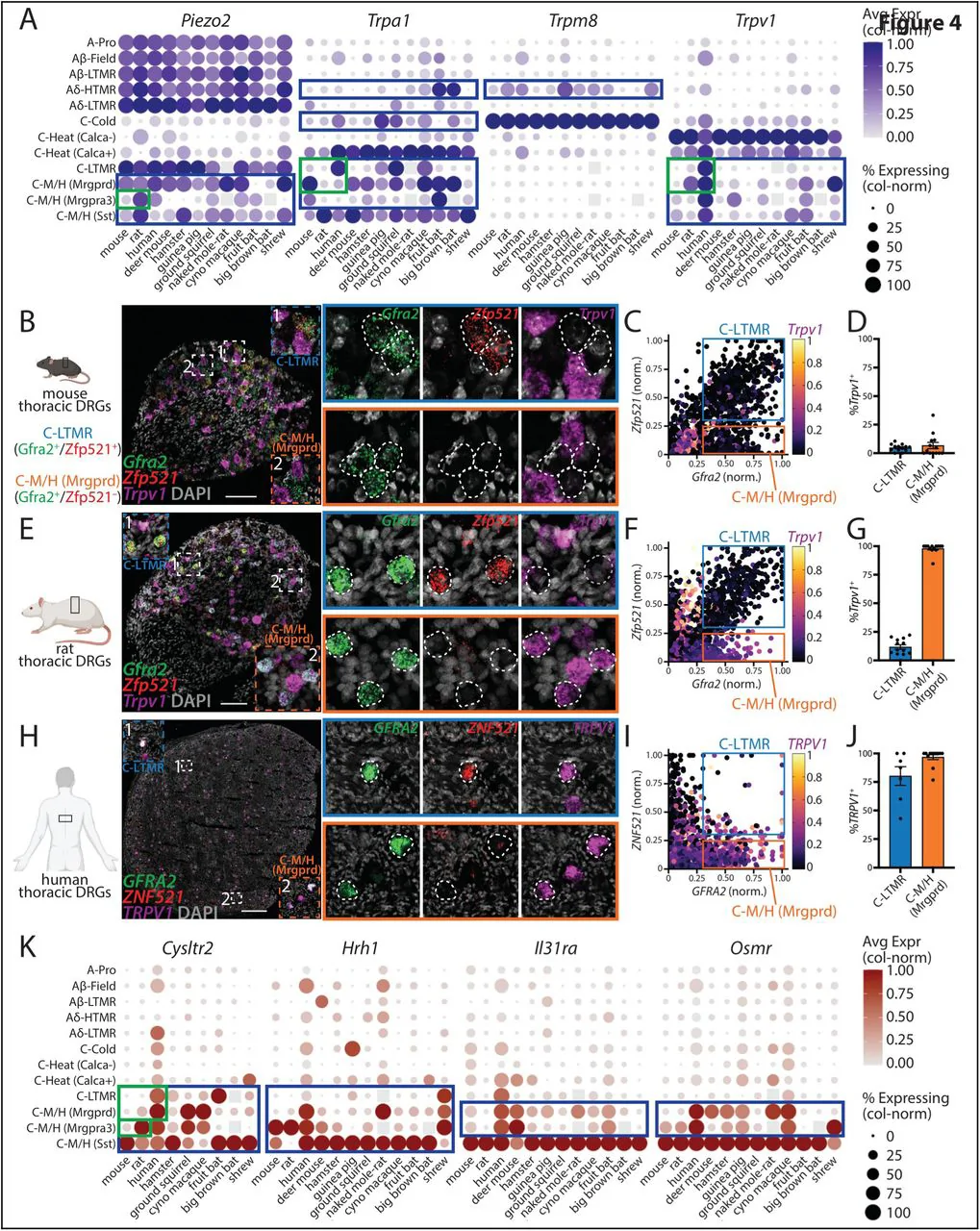
Conserved DRG neuron orthotypes exhibit dynamic sensor/receptor expression patterns across species. (**A**) Dot plot of sensor/receptor expression across conserved orthotypes (rows) and species (columns). Blue boxes indicate orthotype-sensor/receptor pairings exhibiting divergent sensor/receptor expression patterns (shuffling) across species. Green boxes indicate key sensor/receptor expression differences validated by *in situ* hybridization (see panels B to J and fig. S8). Gray boxes, missing cell types (see Fig. 2 and fig. S3). (**B** to **J**) *In situ* hybridization validation of *Trpv1* expression patterns in C-LTMR (*Gfra2*^+^/*Zfp521*^+^) and C-M/H (Mrgprd) (*Gfra2*^+^/*Zfp521*^−^) neurons across mouse (B), rat (E), and human (H) mid-thoracic DRGs. (C), (F), and (I): Quantification of neuronal *Trpv1* expression (2509, 3202, and 2439 neurons from 14, 13, and 12 sections from 6, 6, and 4 DRGs from 3, 3, and 2 mice, rats, and humans, respectively). (D), (G), and (J): Percentages of *Trpv1*^+^ neurons (14, 13, and 12 DRG sections from mice, rats, and humans, respectively); mean ± SEM. Scale bars, 100 μm (B, E), 500 μm (H). (**K**) Dot plot of immune receptor expression across conserved orthotypes (rows) and species (columns). Blue boxes indicate orthotype-receptor pairings exhibiting divergent receptor expression patterns (shuffling) across species. Green boxes indicate key receptor expression differences validated by *in situ* hybridization (fig. S10). Gray boxes, missing cell types (see Fig. 2 and fig. S3).

**Fig. 5.**
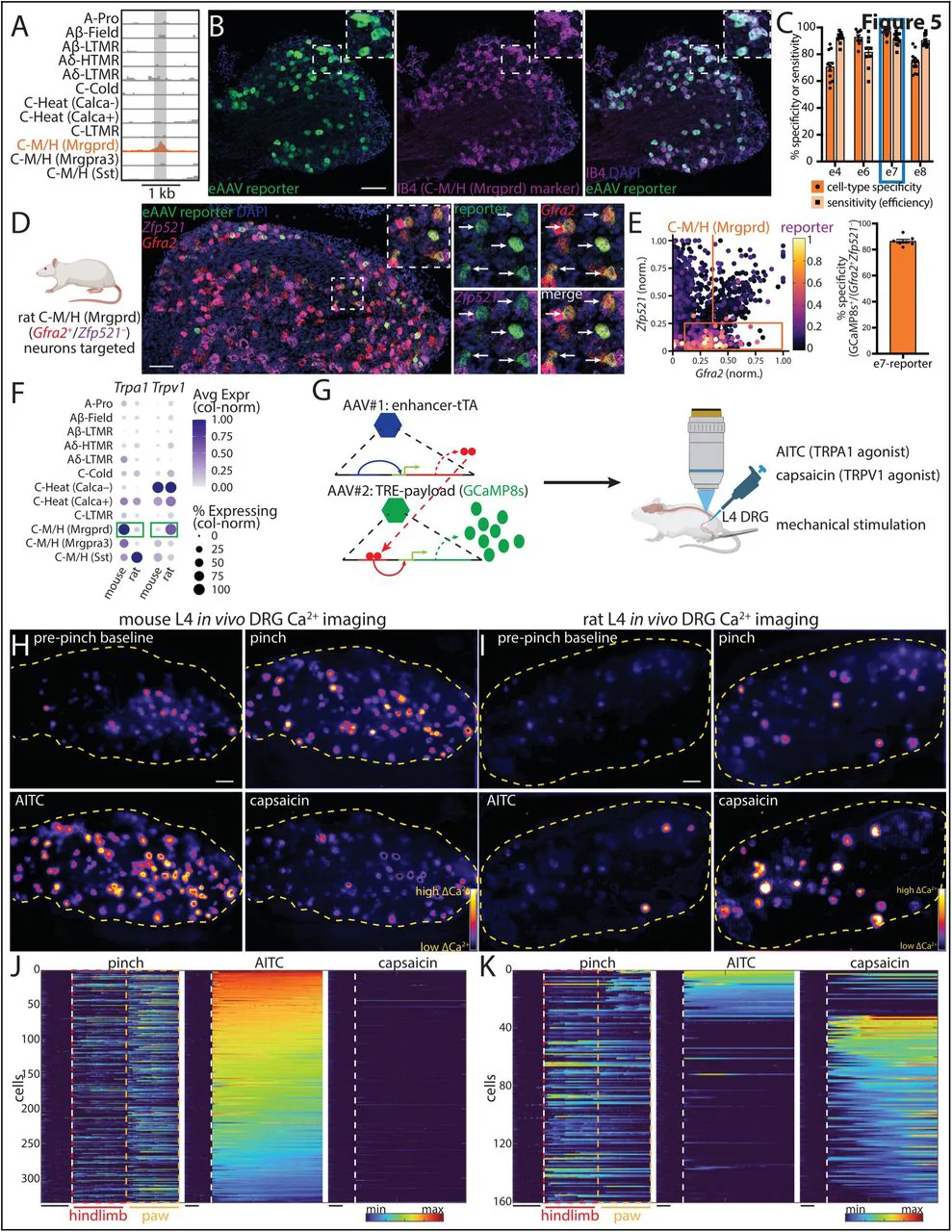
eAAV-enabled *in vivo* Ca^2+^ imaging in mouse and rat reveals C-M/H (Mrgprd) neuron orthotype functional divergence across species. (**A**) Genome tracks showing a candidate cell-type-specific ATAC peak with enhanced accessibility in C-M/H (Mrgprd) neurons (orange). (**B**) Immunohistochemical validation of eAAV payload reporter expression in IB4-binding mouse C-M/H (Mrgprd) neurons. Scale bar, 100 μm. See also figs. S12B and S12C. (**C**) Quantification of specificity and efficiency of C-M/H (Mrgprd) neuron-targeting eAAVs in mice; mean ± SEM. Blue box highlights eAAV tested and used in rats. (**D**) Joint *in situ* hybridization-immunohistochemical validation of eAAV payload reporter expression in *Gfra2*^+^/*Zfp521*^*−*^ rat C-M/H (Mrgprd) neurons. Scale bar, 100 μm. See also fig. S12A. (**E**) Quantification of specificity of C-M/H (Mrgprd) neuron-targeting eAAV in rats; mean ± SEM. (**F**) Single-cell RNA-sequencing data indicating opposite expression patterns of *Trpa1* and *Trpv1* in mouse and rat C-M/H (Mrgprd) neurons. (**G**) Schematic showing eAAV-mediated tTA-TRE-driven GCaMP8s expression in C-M/H (Mrgprd) neurons followed by the *in vivo* Ca^2+^ imaging preparation with mechanical and chemical stimulation. (**H** and **I**) Representative examples of *in vivo* Ca^2+^ imaging fields-of-view of pre-stimulus baselines (upper left) and responses (color bar) to pinch (upper right), AITC (TRPA1 agonist, lower left), and capsaicin (TRPV1 agonist, lower right) in mice (H) and rats (I). Yellow outlines, borders of DRG in field-of-view. Scale bars, 100 μm. (**J** and **K**) Summary heatmaps of Ca^2+^ responses to mechanical (pinch), AITC (TRPA1 agonist), and capsaicin (TRPV1 agonist) stimuli in mice (J) and rats (K). Data are sorted by responsiveness to AITC then capsaicin. N=350 cells, 9 DRGs, 7 mice (J) and 160 cells, 15 DRGs, 10 rats (K). Dotted white lines, stimulus onset. Colored bars, fluorescence (∆F/F). Black bars, 10 seconds.

**Fig. 6.**
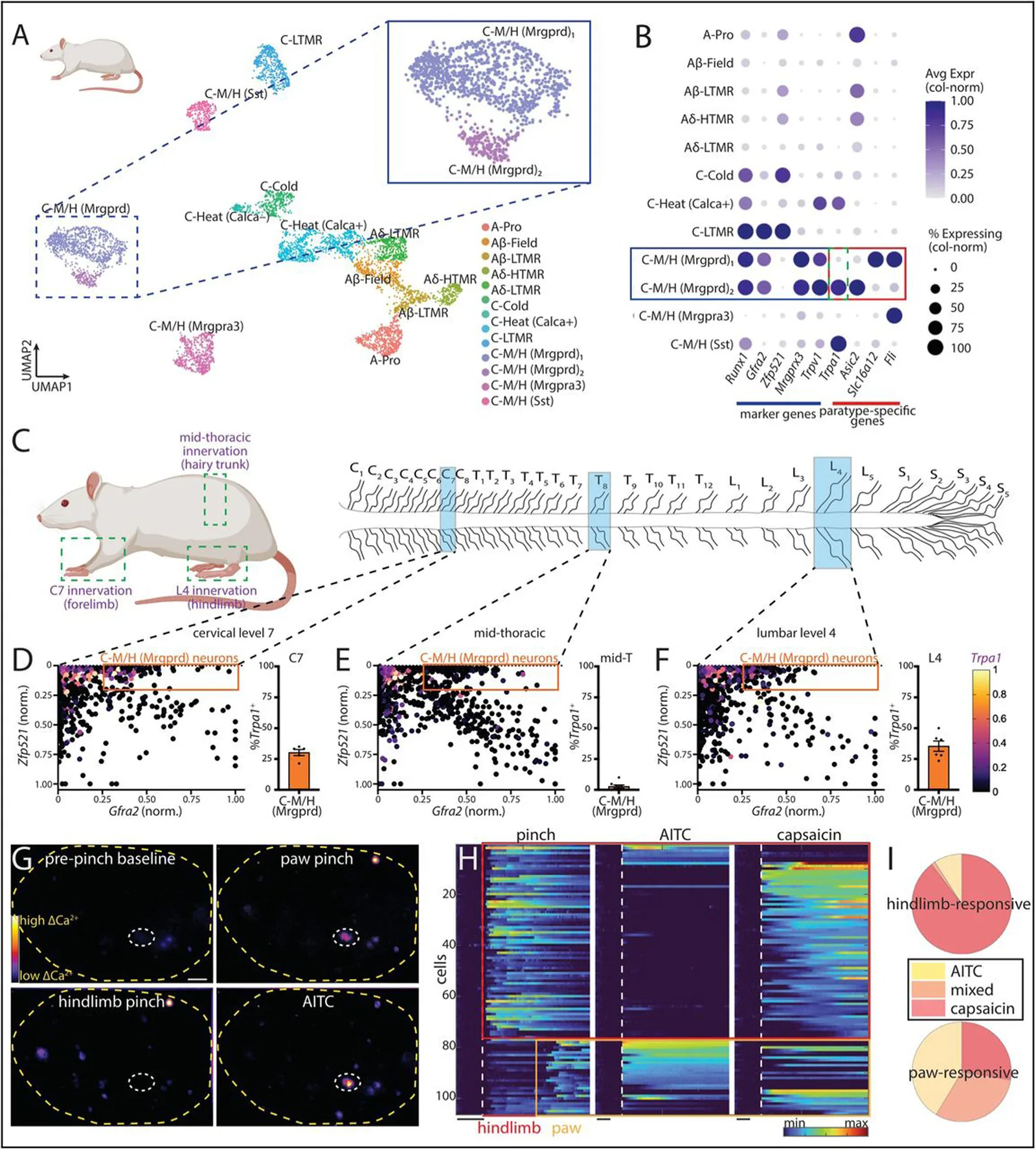
DRG neuron orthotypes diversify into anatomically and functionally distinct species-specific paratypes. (**A**) UMAP embedding of snRNA-seq data from one rat sample. Colors indicate somatosensory neuron orthotype identities. Inset shows splitting of C-M/H (Mrgprd) neuron orthotype into two putative paratypes. (**B**) Dot plot showing expression of common, orthotype-defining marker genes (blue line/box) as well as paratype-defining positive marker genes (red line/box), including *Trpa1* (dotted green box). (**C**) Schematic illustrating innervation of different peripheral cutaneous tissue types by DRGs from different axial levels, including cervical level 7 (C7), mid-thoracic levels (mid-T, e.g., T8), and lumbar level 4 (L4). (**D** to **F**) Quantification of neuronal expression of *Trpa1* by *in situ* hybridization (see fig. S14) in C-M/H (Mrgprd) and other neuron orthotypes in rat C7 (D), mid-T (E), and L4 (F) DRGs. Left, neuronal expression of *Trpa1* (color bar) across all quantified neurons. Right, percentages of *Trpa1*^+^ C-M/H (Mrgprd) neurons; mean ± SEM. (**G**) Representative images of *in vivo* Ca^2+^ imaging fields-of-view of pre-stimulus baseline (upper left) and responses (color bar) to paw pinch (upper right), hairy hindlimb pinch (lower left), and AITC (TRPA1 agonist, lower right). Note overlap between responsive cell signal (dotted white oval) across paw pinch and AITC stimulation. Yellow outline, border of DRG in field-of-view. Scale bar, 100 μm. (**H**) Summary heatmap of Ca^2+^ responses to hairy hindlimb (red box) and glabrous paw (yellow box) pinch-responsive neurons and their responses to AITC (TRPA1 agonist) and capsaicin (TRPV1 agonist). N = 106 cells, 15 DRGs, 10 rats. Traces are sorted by responsiveness to hindlimb pinch, then paw pinch, then AITC, then capsaicin. Dotted white lines, stimulus onset. Colored bar, fluorescence (∆F/F). Black bars, 10 seconds. (**I**) Pie charts showing percentage of hairy hindlimb- and glabrous paw-responsive neurons that also respond to AITC (yellow), capsaicin (red), or both (orange).

## Data Availability

Further information and requests for resources and reagents should be directed to David Ginty (David_Ginty@hms.harvard.edu). All unique reagents generated in this study will be made available upon reasonable request. The original sequencing data will be deposited to the Gene Expression Omnibus (GEO: XXXXXXX) and made public upon publication of the manuscript.
