# Supplementary material for: The functional diversification of somatosensory neuron repertoires across Mammalia
Source: bioRxiv. 2026 Sep 21:2026.09.19.752904. Preprint. [Version 1] doi: 10.64898/2026.09.19.752904 (PMC13622097; doi:10.64898/2026.09.19.752904)
Supplement: Supplementary file 1 [file NIHPP2026.09.19.752904v1-supplement-1.pdf]

bioRxiv preprint doi: https://doi.org/10.64898/2026.09.19.752904; this version posted September 21, 2026. The copyright holder for this preprint (which was not certified by peer review) is the author/funder, who has granted bioRxiv a license to display the preprint in perpetuity. It is made available under a CC-BY 4.0 International license.

Supplementary Materials

Materials and Methods

Figs. S1 to S16

References

Movies S1 to S2

Materials and Methods

Animals

All experiments performed in this study were approved by the Institutional Animal Care and Use Committee of Harvard Medical School. Experiments followed the ethical guidelines outlined in the NIH Guide for the care and use of laboratory animals (https://grants.nih.gov/grants/olaw/guide-for-the-care-and-use-of-laboratory-animals.pdf). C57BL/6 male and female mice (*Mus musculus*; C57BL/6J, The Jackson Laboratory, RRID:IMSR_JAX:000664) were used for sequencing and RNAscope in situ hybridization studies. CD1 male and female mice (Crl:CD1(ICR), Charles River, RRID:IMSR_CRL:022) were used for eAAV screening and eAAV-mediated physiological and behavioral experiments. Mice used for physiology and behavioral experiments were 4 – 8 weeks old. Sprague-Dawley male and female rats (*Rattus norvegicus*; Charles River, RRID:RGD_737891) were used for all rat experiments. Rats used for physiology and behavioral experiments were ~3 and 4 – 8 weeks old, respectively. All mice and rats used for sequencing experiments were of weaning age (postnatal days 21 - 24). Roughly equal numbers of rodents of both sexes were used across all experiments. Mice and rats were housed in a temperature-controlled and humidity-controlled facility, maintained on a 12 h light/dark cycle, and given food and water ad libitum.

Tissue from rats (*Rattus norvegicus*; Charles River, RRID:RGD_737891), deer mice (*Peromyscus maniculatus bairdii*; Harvard University), hamsters (*Mesocricetus auratus*; Charles River/Massachusetts Institute of Technology, RRID:CVCL_TQ39), naked mole-rats (*Heterocephalus glaber*; Columbia University), ground squirrels (*Ictidomys tridecemlineatus*; Yale University), bats (*Rousettus aegyptiacus*, *Eptesicus fuscus*; Johns Hopkins University), and Etruscan shrews (*Suncus etruscus*; Humboldt University of Berlin) used for single-nucleus sequencing experiments were obtained in the laboratories of Drs. S. Hui (rat), H. Hoekstra (deer mouse), S. Hrvatin (hamster), I. Abdus-Saboor (naked mole-rat), E. O. Gracheva (ground squirrel), C.F. Moss (fruit bat, big brown bat), and M. Brecht (Etruscan shrew). Single-nucleus preparation was performed at Harvard Medical School for all samples except the naked mole-rat samples; single-nucleus preparation for these samples was performed at Columbia University.

DRG Single-nucleus RNA- and ATAC-sequencing

We collected two to four biological replicates from distinct individuals for each species, including at least one male and one female whenever possible. Most samples were collected from healthy weanling or young adult animals. All rodent samples except the naked mole-rat samples were collected from healthy weanlings. DRGs from all axial levels were rapidly dissected out in DMEM-F12, HEPES (Thermo Fisher/Gibco, 11330-032; supplemented with 12.5 mM D-glucose and 1x pen/strep) on ice. DRGs were then snap frozen and stored at -80˚C until further use or immediately processed for nuclear extraction and production of single-nucleus suspensions.

To produce DRG single-nucleus suspensions, DRGs were transferred to homogenization buffer (0.25 M sucrose, 25 mM KCl, 5 mM $MgCl_2$, 20 mM Tricine-KOH, pH 7.8), dounced, and then layered onto OptiPrep gradients (OptiPrep™ Density Gradient Medium, Sigma-Aldrich, D1556-250ML). Various additives were included in homogenization and Optiprep/iodixanol solutions: 1 mM DTT (Life Technologies, 707265ML), 150 µM spermine-tetrachloride (spermine-tetrahydrochloride, Sigma-Aldrich, S1141-1G), 500 µM spermidine-trichloride (spermidine-trihydrochloride, Sigma-Aldrich, S2501-1G),

bioRxiv preprint doi: https://doi.org/10.64898/2026.09.19.752904; this version posted September 21, 2026. The copyright holder for this preprint (which was not certified by peer review) is the author/funder, who has granted bioRxiv a license to display the preprint in perpetuity. It is made available under a CC-BY 4.0 International license.

protease inhibitor cocktail (Roche/Millipore Sigma, 11873580001; 100X stock made by dissolving two protease inhibitor tablets in 1 mL nuclease-free water), 10 mM sodium butyrate (Sigma, 19-137), and RNase inhibitor (Protector RNase inhibitor, Sigma, 3335402001; 1 U/µL for Multiome and 0.2 U/µL for 3'GEX). After ultracentrifugation (3,000 g for 15 minutes with no brake in a swinging bucket centrifuge at 4˚C), the 30%–40% interface was collected, diluted and passed through a 40-µm filter. Nuclei were washed, pelleted, washed, and resuspended. Nuclei collected for Multiome (RNA and ATAC) samples were then processed for ATAC transposition, loaded onto 10x Genomics Chip J chips, and processed according to 10x Genomics Multiome protocol, while nuclei collected for 3'GEX (RNA only) samples were loaded onto 10x Genomics Chip G chips and processed according to 10x Genomics 3'GEX protocol.

10x Genomics protocols were followed to generate Multiome (RNA and ATAC) or 3'GEX (RNA only) libraries. Libraries were sequenced on NextSeq500, NovaSeq6000, or NovaSeq X Plus platforms, demultiplexed, and mapped to the appropriate reference genomes custom built using 10x Genomics pipelines. Reference genomes used for mapping included mm10 (mouse), mRatBN7 (rat), HU_Pman_2.1 (deer mouse), BCM_Maur_2 (hamster), HiC_Itri_2 (ground squirrel), Naked_mole-rat_maternal (naked mole-rat), mRouAeg1 (fruit bat), mEptFus1 (big brown bat), and mSunEtr1 (Etruscan shrew). Samples were sequenced to ≥19,979 mean reads per cell. After mapping to the appropriate reference genomes, we obtained new transcriptomes for a total of 59,200 (mouse), 108,194 (rat), 41,648 (deer mouse), 61,013 (hamster), 35,240 (ground squirrel), 66,507 (naked mole-rat), 86,131 (fruit bat), 72,450 (big brown bat), and 15,717 (Etruscan shrew) DRG nuclei, with a range of 15,717 – 108,194 cells across species and a mean of 60,678 cells per species. Mean reads per cell for each library: 51,327, 53,779, 38,565, 35,910 (mouse); 24,213, 27,899, 21,350 (rat); 31,141, 20,815 (deer mouse); 20,945, 24,850 (hamster); 25,296, 40,453 (ground squirrel); 26,132, 22,935, 19,979 (naked mole-rat); 23,763, 40,470, 23,262, 23,583, 49,672 (fruit bat); 26,026, 21,860 (big brown bat); 32,956, 90,527 (shrew). Median genes per cell for each library: 1,603, 1,228, 1,285, 801 (mouse); 1,274, 1,238, 1,358 (rat); 921, 745 (deer mouse); 1,695, 1,944 (hamster); 1,578, 707 (ground squirrel); 1,011, 833, 944 (naked mole-rat); 1,544, 477, 848, 1,161, 771 (fruit bat); 1,155, 951 (big brown bat); 822, 807 (shrew). Median genes per high-quality DRG neuron library were substantially higher than these per-cell averages (fig. S1). We integrated analysis of the above samples with analysis of samples previously collected from guinea pig (*Cavia porcellus*) and cynomolgus macaque (*Macaca fascicularis*) (*33*), rhesus macaque (*Macaca mulatta*) (*34*), and human (*Homo sapiens*) .

Data were processed using Seurat (*108*). Briefly, standard quality control metrics were applied. Data were then normalized, highly variable genes identified, and dimensional reduction and clustering performed. Clusters expressing non-neuronal gene markers were removed (*17*, *18*), leaving only neuronal populations. Clusters were annotated by expression of key conserved cell-type-specific genes (*13*, *17*, *33*, *34*, *39*, *40*) and validated using cross-species integration approaches.

Single-nucleus Transcriptome Analysis

Standard quality control, analysis, and orthotype annotation

To extract high-quality neuronal data and determine the neuronal cell types constituent of each species' DRG somatosensory neuron repertoires, we followed a standard quality control and cell-type annotation pipeline. For each sample, the single-nucleus RNA-seq count matrix was loaded from Cell Ranger output and initially filtered to retain high-quality nuclei. Gene expression was normalized using SCTransform, followed by Principal Component Analysis (PCA), Uniform Manifold Approximation and Projection (UMAP) embedding, and graph-based Leiden clustering (30 principal components, resolution 0.5); non-neuronal clusters were then removed and the normalization, dimensionality-reduction, UMAP embedding, and clustering steps were repeated on the retained nuclei, with canonical marker genes used to confirm neuronal identity. This process was repeated iteratively to obtain high-quality neuronal clusters (fig. S1).

Cross-species integration

To enable direct cross-species transcriptomic comparisons, scRNA-seq count matrices from all non-mouse species were first converted to mouse gene symbols. Ortholog mapping was predominantly executed using

bioRxiv preprint doi: https://doi.org/10.64898/2026.09.19.752904; this version posted September 21, 2026. The copyright holder for this preprint (which was not certified by peer review) is the author/funder, who has granted bioRxiv a license to display the preprint in perpetuity. It is made available under a CC-BY 4.0 International license.

NCBI gene ortholog databases (*109*), supplemented with Ensembl BioMart exports for the naked mole-rat and guinea pig (*110*). Only 1:1 orthologs to mouse were retained for cross-species integration.

Downstream processing, clustering, and integration were conducted using Seurat. Quality control protocols filtered out low-quality cells, retaining only those with a minimum feature count (e.g., >1,500 detected genes) and a mitochondrial read percentage below 20%. Furthermore, human nuclei were specifically filtered to require MALAT1 expression greater than 2 (*111*). Following filtration, gene counts were log-normalized, and the top 2,000 highly variable features were identified independently for each species-specific dataset. To correct for batch and species-specific effects, datasets were integrated using Canonical Correlation Analysis via Seurat's anchor-based integration framework. The integrated assay was scaled and subjected to PCA. Principal components (PCs) that cumulatively captured 90% of the variance, where no subsequent PC contributed more than 5%, were selected to construct a nearest-neighbor graph. Finally, cells were clustered using a graph-based approach (resolution = 3.0) and embedded into two-dimensional space using t-SNE and UMAP, with cell populations validated via the projection of established neuronal and non-neuronal marker genes. Clusters were reannotated based on marker gene expression profiles.

ShinyCell was used to build an interactive web resource to share the complete cross-species integrated object with the field (*112*).

Within-species biological replicate analysis

To test for consistency between biological replicates, we collected samples from distinct individual animals of the same species and merged the resulting datasets. Single-nucleus RNA-seq replicates of interest were loaded, labeled by their dataset of origin, and merged into a single object using raw RNA counts via the Seurat merge function. The merged, un-integrated data were then log-normalized, reduced to the top 2,000 variable features, scaled, and embedded by PCA and UMAP (30 principal components), and the resulting UMAP was visualized and colored by both replicate/dataset of origin and annotated with orthotype labels to assess data quality and consistency across replicates (fig. S2, A to F). Integration approaches were avoided to ensure high sensitivity to batch effects, technical noise, and/or biological variability.

Cross-species gene expression analyses (gene expression heatmap and dotplots)

For cross-species gene expression analyses, two major approaches to visualize data were taken. To display the relative expression levels of conserved orthotype marker genes, we generated a cross-species marker gene heatmap. For each sample, mean expression of the selected genes was computed per orthotype from the corresponding Seurat object, with gene names matched case-insensitively and genes absent from a given dataset recorded as missing values. Expression values were then min–max normalized to a 0 – 1 scale within each sample and gene and visualized as a heatmap of normalized expression across cell types and samples, with missing genes shown in gray. To display the relative expression levels of smaller numbers of sensor, immune receptor, transcription factor, growth factor receptor, and ion channel genes, we generated cross-species plots. For each sample, the selected genes (matched case-insensitively) were extracted from the corresponding Seurat object, and for each orthotype the mean expression and/or percentage of cells with detectable expression were computed. Within each sample and gene, both the mean expression and percent-expressing values were min–max normalized to a 0 – 1 scale across cell types and displayed faceted by gene, with point color encoding normalized mean expression and point size encoding the normalized percentage of expressing cells.

Orthotype whole-transcriptome orthology tree analyses

To ask if certain DRG neuron types are generally more variable across Mammalia, we sought to describe the relative rates at which the whole transcriptomes of different DRG neuron orthotypes have diverged rather than relying on the differential expression of a small number of sensor and receptor genes. To do so, we assembled transcriptome distance trees, a measure of transcriptome evolution, for each orthotype by performing Spearman rank-correlations on cell-type averaged (pseudobulk) transcriptome measurements.

bioRxiv preprint doi: https://doi.org/10.64898/2026.09.19.752904; this version posted September 21, 2026. The copyright holder for this preprint (which was not certified by peer review) is the author/funder, who has granted bioRxiv a license to display the preprint in perpetuity. It is made available under a CC-BY 4.0 International license.

One-to-one orthologs across five rodent species (mouse, rat, deer mouse, hamster, and squirrel) that share all major DRG neuron orthotyeps were obtained from a mouse-referenced BioMart ortholog table, retaining only genes annotated as one-to-one orthologs in every species and yielding ~12,984 unique orthologous gene sets. For each single-nucleus RNA-seq replicate, gene identifiers were converted to their mouse reference symbols via case-insensitive matching, datasets were subset to the ~10,060 genes shared across all objects, and counts were pseudobulked by summing raw counts within each replicate × orthotype before normalizing to log-CPM. To construct transcriptome-based trees for individual orthotypes, the corresponding pseudobulk columns were variance-filtered, z-scored across samples, and converted to a distance matrix of 1 – Spearman's rho (ρ) between samples, from which neighbor-joining trees were built; branch support was estimated by gene-level bootstrapping (1,000 resamples of genes with replacement), with clade support computed as the proportion of bootstrap trees recovering each clade. To quantify inter-orthotype transcriptome divergence, replicates were collapsed by species (summing raw counts within each species × cell type, then re-normalizing to log-CPM), and for each orthotype a cross-species divergence metric was computed as the mean pairwise distance (1 – ρ) across the five species. Uncertainty in this metric was assessed by gene bootstrapping (1,000 resamples), and per-orthotype divergence distributions were summarized as bootstrap quantile boxplots. C-Heat (Calca–) neurons were excluded from subsequent inter-orthotype analyses due to their extremely low cell numbers (fig. S3).

To measure inter-orthotype variability in transcriptomic divergence across species, we summed total tree branch lengths for each orthotype. These values represent quantitative measures of pseudobulk transcriptome distance or similarity that can be plotted on a tree to display pseudobulk transcriptome measures of relatedness. Notably, these trees did not necessarily recapitulate known species phylogeny (fig. S11B), rather suggesting substantial interspecies dynamicity in gene expression such that each species may adopt orthotype-characteristic transcriptomic profiles. This result suggests that some orthotypes have undergone significant transcriptomic divergence between some mammalian species relative to others. By contrast, the phylogenetic recapitulation exhibited by this analysis performed on spermatogonial cells suggests that distinct cell lineages may differ with regards to whether transcriptome distances recapitulate organismal phylogeny or not (*78*).

Single-nucleus ATAC-seq Analysis

Single-nucleus ATAC-seq data were processed in R using Seurat (*108*) and Signac (*113*) packages.

Single-nucleus ATAC-sequencing UMAP embeddings

To generate single-nucleus ATAC-seq UMAP embeddings for each of several rodent samples, the Cell Ranger ARC peak-by-cell matrix and fragment file were loaded into a Signac chromatin assay annotated with Ensembl gene models, filtered to previously identified neuronal barcodes with transferred cell-type labels, and quality-filtered on ATAC counts, nucleosome signal, and TSS enrichment. Accessibility was then normalized by TF-IDF, reduced by latent semantic indexing (SVD on the top features), and embedded and clustered using the LSI dimensions (UMAP and graph-based Leiden clustering), after which non-neuronal or low-quality clusters were removed and the normalization, dimensionality-reduction, and clustering steps were repeated on the retained cells before UMAP embeddings were plotted.

ChromVAR analysis

For each of five rodent species (mouse, rat, deer mouse, hamster, and ground squirrel) that share all major DRG neuron orthotyeps, neuronal nuclei passing an initial fragment threshold (>500 ATAC fragments) were retained based on barcodes from the matched single-cell RNA-seq objects, fragments were quantified against the per-sample Cell Ranger ARC peak set (after removing peaks <20 bp or >10 kb), and cell-type labels were transferred from the RNA objects. Peaks were then re-called for each cell type using MACS3 and quantified into a new chromatin assay; for several species, peak coordinates were remapped from RefSeq accessions to the chromosome/scaffold names of the corresponding BSgenome and filtered to sequences within genome bounds, with mouse peaks additionally restricted to standard chromosomes and depleted of ENCODE blacklist regions. Per-base sequence statistics and JASPAR2020 vertebrate CORE

bioRxiv preprint doi: https://doi.org/10.64898/2026.09.19.752904; this version posted September 21, 2026. The copyright holder for this preprint (which was not certified by peer review) is the author/funder, who has granted bioRxiv a license to display the preprint in perpetuity. It is made available under a CC-BY 4.0 International license.

motif matches were added to each object, and per-cell transcription factor (TF) motif accessibility was estimated using chromVAR. To compare motif activity across species, chromVAR deviation scores were collapsed into TF families (by normalizing TF names and stripping numeric and letter suffixes), family-level scores were averaged per cell type, and families were ranked by their variance across cell types in the mouse dataset to select the ten most variable families. For visualization, family-averaged chromVAR scores were min–max normalized within each sample and family across cell types and displayed as a dot plot faceted by TF family, with point color encoding normalized mean deviation and point size encoding the normalized fraction of cells with positive deviation, plotted for all five species side by side.

Signac LinkedPeaks analysis

Mouse and rat sensor gene-peak linkage analysis

For each species, peak sets called by Cell Ranger ARC across samples were merged into a unified set of genomic ranges, and peaks shorter than 20 bp or longer than 10 kb were removed; fragments were then re-quantified against this common peak set in every sample. Nuclei were filtered to retain those with more than 500 ATAC fragments and to keep only neuronal barcodes previously identified in the corresponding single-nucleus RNA-seq objects, and the matched RNA and SCT assays together with their cell-type annotations were transferred onto the ATAC objects before all samples of a species were merged into a combined object. Peaks were then re-called per cell type on the merged data using MACS3, retaining only standard chromosomes and (for mouse) excluding ENCODE blacklist regions, and fragment counts were quantified against this refined peak set to build a new chromatin assay annotated with Ensembl gene models (mouse: EnsDb.Mmusculus.v79 with UCSC-style chromosome names; rat: EnsDb.Rnorvegicus.v105 with NCBI-style names). The resulting per-species objects, with cell types set as the active identities, were retained for downstream analyses.

For each species, peak GC content was computed and peak-to-gene links were identified for the sensor gene by correlating peak accessibility with SCT-normalized gene expression within a 500 kb window upstream and downstream of the gene (LinkPeaks, score cutoff 0.01). The resulting links and accessibility profiles were visualized per orthotype as coverage plots spanning the same flanking window. Linked peaks from one species were mapped to corresponding genomic loci/coordinates in the other species using the UCSC LiftOver online browser tool. The homologous coordinates were then replotted on coverage plots spanning the locus and 2 kb flanking windows.

Histology: In Situ Hybridization (RNAscope) and Immunohistochemistry.

Unless stated otherwise, mid-thoracic (T6 – T10, usually T8) DRGs were collected and used in studies contributing to Figs. 2 to 4 and figs. S4 to S10; lumbar level 4 (L4) DRGs were collected and used in studies contributing to Fig. 5 and fig. S12; and cervical level 7 (C7), mid-thoracic, and lumbar level 4 (L4) DRGs were collected and used in studies contributing to Fig. 6 and figs. S14 and S15.

In situ hybridization (ISH)

Individual DRGs from rodents were rapidly dissected out in DMEM-F12, HEPES (Thermo Fisher/Gibco, 11330-032; supplemented with 12.5 mM D-glucose and 1x pen/strep) on ice. Axial levels were identified using the T12 DRG as a landmark: the T12 DRG was defined as the ganglion immediately caudal to the last rib. Macaque thoracic (T8-T10) DRGs were obtained from the Oregon National Primate Research Center. Human thoracic (T8-T10) DRGs were obtained from AnaBios. DRGs were embedded in OCT (Fisher, 1437365) on ice, flash-frozen, and stored at −80 °C until sectioning on a cryostat. DRGs were sectioned at a thickness of 20 – 25 µm. Target transcripts were detected by RNAscope (Advanced Cell Diagnostics) using the manufacturer's protocol. Briefly, after cryosectioning, slides were fixed in 4% prechilled PFA (paraformaldehyde; Sigma, P6148-500G, or EMS 32% aqueous solution, 15714-S) at 4°C for 15 mins, then dehydrated using serial concentrations of ethanol. Next the sections were digested using Protease Plus for 10 mins at RT. After washing, a mix of RNAscope probes was added on the slide and incubated at 40°C for 2 – 4 hours, followed by sequential AMP1, AMP2, and AMP3 steps, according to the protocol. Slides were

bioRxiv preprint doi: https://doi.org/10.64898/2026.09.19.752904; this version posted September 21, 2026. The copyright holder for this preprint (which was not certified by peer review) is the author/funder, who has granted bioRxiv a license to display the preprint in perpetuity. It is made available under a CC-BY 4.0 International license.

mounted in DAPI Fluoromount-G (Southern Biotech, 0100-20) and imaged on a Zeiss LSM 900 confocal microscope.

The following RNAscope probes were used: Mm-Gfra2 (441481-C2), Mm-Rbfox3 (313311-C3), Mm-Th (317621-C3), Mm-Zfp521 (565831), Rn-Gfra2 (463041), Rn-Rbfox3 (436351-C3), Rn-Zfp521 (1299151-C2), Hg-GFRA2 (1299141-C2), Hg-RBFOX3 (541911-C3), Hg-ZNF521 (1299121-C1), Mfa-GFRA2 (466311), Mmu-RBFOX3 (870421-C3), Mmu-ZNF521 (1333631-C2), Hs-GFRA2 (463011), Hs-RBFOX3 (415591-C2), Hs-ZNF521 (565741-C3).

Mm-Trpa1 (400211-C3), Mm-Trpv1 (313331-C3), Mm-Cysltr2 (452621-C3), Mm-Etv1 (433281-C2), Mm-Gfra1 (431781), Mm-Piezo2 (400191-C3), Mm-Avil (498531), Mm-Mrgpra3 (548161-C3), Mm-Mrgprb4 (435781-C2), Rn-Trpa1 (312511-C3), Rn-Trpv1 (501161-C3), Rn-Cysltr2-O1 (1575841-C3), Rn-Etv1 (1209241-C1), Rn-Gfra1 (463031-C2), Rn-Piezo2 (549741-C3), Hs-TRPA1 (311951-C2), Hs-CYSLTR2 (543481-C2)

Cell-type proportion quantifications:

For the data in Fig. 2 and fig. S4F, total numbers of neurons per DRG section were recorded by counting *Rbfox3*$^{+}$/*RBFOX3*$^{+}$ cells. Hair follicle density estimates from were derived from the literature (*53*, *54*). For the data in fig. S15, total numbers of neurons per DRG section were recorded by counting *Avil*$^{+}$ cells.

Sensor/receptor-cell type quantifications:

To quantify sensor/receptor expression in distinct orthotypes using ISH data, we manually outlined individual DRG neuron cell bodies in Fiji (ImageJ; NIH, https://imagej.net/Fiji/Downloads) and measured the mean fluorescence level of all three channels (marker gene 1, marker gene 2, and sensor/receptor) in each of tens to hundreds of cells per section. Normalized signal from all channels was then quantified and three-dimensional results plotted using Prism (Prism 11, GraphPad, RRID:SCR_002798, https://www.graphpad.com/), with co-marker gene 1 on the x-axis, co-marker gene 2 on the y-axis, and the sensor/receptor fluorescence levels plotted by color. Individual marker gene fluorescence level distributions were examined and thresholds for demarcating cell types based on marker genes were determined. Finally, sensor/receptor fluorescence level distributions were examined and thresholds for defining a cell as sensor/receptor-positive versus -negative were determined.

Immunohistochemistry (IHC):

Animals were perfused with PBS followed by 4% PFA. Spinal columns were removed and DRGs and/or spinal cords dissected out. Back hairy skin was removed, treated with Nair hair removal lotion (NAIR hair removal cream, Church and Dwight Co., Princeton, NJ), and gently rubbed to remove hair and underlying fat. Skin was then washed and fixed in Zamboni fixative (picric acid-formaldehyde (PAF) fixative, Fisher, NC9335034) overnight (4°C). Tissue was embedded in OCT (Fisher, 1437365) or NEG-50, frozen and stored at –80°C. Tissue was cryosectioned and mounted on Superfrost plus slides (Fisher, 12-550-15). After drying at room temperature (RT) overnight, slides were rehydrated and washed with PBS, and blocked by 5% Normal Donkey Serum (Jackson, 017-000-121) for 1 – 2 hours at RT. Then primary antibody mix was added overnight at 4°C. Following incubation with primary antibody, slides were washed with 0.1% PBS-Triton, the secondary antibody mixed was added for 2 hours at RT. Finally, slides were washed in PBS, mounted in DAPI Fluoromount-G (Southern Biotech, 0100-20), and imaged using a confocal microscope (Zeiss LSM 900).

Primary antibodies used were goat anti-mCherry (Origene, AB0040-200; RRID:AB_2333093; 1:500), goat anti-GFP (US Biological Life Sciences, G8965-01E; 1:500), rabbit anti-TH (Sigma, AB152; RRID:AB_390204; 1:500), and rabbit anti-CGRP (Immunostar, 24112; RRID:AB_572217; 1:500). IB4-fluorophore conjugates (IB4, Alexa Fluor 647 conjugated; Thermo Fisher, I32450) were used at 1:500. Secondary antibodies included Donkey anti-goat (Alexa Fluor 488) (Thermo Fisher, A-11055; RRID:AB_2534102), Donkey anti-goat (Alexa Fluor 546) (Thermo Fisher, A-11056; RRID:AB_2534103),

bioRxiv preprint doi: https://doi.org/10.64898/2026.09.19.752904; this version posted September 21, 2026. The copyright holder for this preprint (which was not certified by peer review) is the author/funder, who has granted bioRxiv a license to display the preprint in perpetuity. It is made available under a CC-BY 4.0 International license.

Donkey anti-rabbit (Alexa Fluor 488) (Thermo Fisher, A-21206; RRID:AB_2535792), and Donkey anti-rabbit (Alexa Fluor 546) (Thermo Fisher, A-10040; RRID:AB_2534016).

In situ hybridization-immunohistochemistry (ISH-IHC):

To accommodate joint in situ hybridization and immunohistochemistry on the same tissue sections, we performed the ISH protocol described above followed by the IHC protocol described above.

Enhancer AAV (eAAV) Screening

Single-nucleus multiome ATAC-seq dataset processing and cell-type-specific enhancer nomination (PIASO pipeline)

Mouse DRG single-nucleus multiome ATAC-seq data were processed with PIASO (*114*) (https://github.com/genecell/PIASO). ATAC fragments were imported into a Cytome database file using piaso.pp.importFragments with per-nucleus transcription start site (TSS) enrichment computed inline against the mm10 TSS annotation; nuclei were retained if they had ≥ 1,000 fragments and a TSS enrichment ≥ 3, yielding 19,721 nuclei. Groups for peak calling were defined by two-level local Leiden clustering: a coarse partition (piaso.tl.leiden, resolution 0.25) followed by within-group refinement (piaso.tl.leiden_local, resolution 0.75), the latter re-embedding each coarse group in its own term frequency–inverse document frequency (TF-IDF) normalization (piaso.tl.run_TFIDF) followed by randomized singular value decomposition (SVD; 30 components). Peak calling was then performed by PICCO (piaso.tl.picco), operating directly on the compressed fragment store in Cytome. Peaks were quantified into a cell × peak count matrix (piaso.pp.quantifyPeakActivity) and TF-IDF-normalized. Cell-type labels were transferred from the independent analysis of the paired snRNA-seq multiome data, matched by cell barcode. Cell type-specific enhancers were nominated with COSG (*115*) applied to the TF-IDF peak layer (cosg.run_cosg_cytome), grouped by the RNA-derived cell-type annotation (mu = 100; expressed_pct = 0.05). From the ranked enhancer-candidate lists produced by COSG, we manually inspected the per-cell-type peak distributions in the corresponding bigWig tracks and selected the genomic regions surrounding the peak summits. To further prioritize candidates, we incorporated vertebrate sequence conservation, computed with phastCons (*116*, *117*) from the 60-way vertebrate alignment (mm10.60way.phastCons.bw, downloaded from http://hgdownload.cse.ucsc.edu/goldenpath/mm10/phastCons60way/).

AAV production and neonatal IP injection

AAV genome plasmids were constructed using standard cloning and molecular biology techniques (constructs generated in this paper: pAAV-mBG-mScarlet; pAAV-mBG-ReaChRmCitrine; pAAV-TRE3g-GCaMP8s-WPRE-bGHpA). AAVs (serotype PHP.S) were packaged and concentrated using transient transfection of PHP.S, pHelper, and AAV-genome plasmids into HEK 293T cells. Supernatants were collected at 72 and 120 hours post-transfection. At 120 hours post-transfection, cells were scraped off plates and collected. 293T cell pellets were extracted using a lysis buffer containing salt active nuclease (ArcticZymes) in 40 mM Tris, 500 mM NaCl and 2 mM $MgCl_2$ pH 8 (SAN buffer). Viral supernatants were concentrated via 8% PEG/500 mM NaCl precipitation and re-suspended in SAN buffer. Cleared viral lysates were then loaded onto an iodixanol gradient (Optiprep; OptiPrep™ Density Gradient Medium, Sigma-Aldrich, D1556-250ML), centrifuged, and concentrated using Amicon filters with a 100-kD molecular cutoff to a final volume of approximately 15 – 20 µL. AAVs were injected intraperitoneally into pups at postnatal days 0 – 2.

eAAV injections

For eAAV screening, 5e11 – 2e12 genome copies (GC) per eAAV were injected into mouse pups. For eAAV-mediated behavioral testing, 2e11 – 4e12 GC eAAV(PHP.S)-C-LTMRe1L-ReaChR-mCitrine and eAAV(PHP.S)-C-LTMRe1L-mScarlet was injected into mouse pups, and 2e12 – 4e13 GC eAAV(PHP.S)-C-LTMRe1L-ReaChR-mCitrine and eAAV(PHP.S)-C-LTMRe1L-mScarlet was injected into rat pups. For eAAV-mediated Ca^{2+} imaging physiology, 5e11 – 1e12 GC eAAV(PHP.S)-C-M/H(Mrgprd)e7-tTA was

bioRxiv preprint doi: https://doi.org/10.64898/2026.09.19.752904; this version posted September 21, 2026. The copyright holder for this preprint (which was not certified by peer review) is the author/funder, who has granted bioRxiv a license to display the preprint in perpetuity. It is made available under a CC-BY 4.0 International license.

injected along with 1e12 GC AAV(PHP.S)-TRE-GCaMP8s into mouse pups, while 1e12 – 2e12 GC eAAV(PHP.S)-C-M/H(Mrgprd)e7-tTA was injected along with 1e12 – 2e12 GC AAV(PHP.S)-TRE-GCaMP8s into rat pups.

Behavioral Analyses

Animals

Mice and rats were maintained on a standard 12-hour light/dark cycle (lights on 7:00 AM, lights off 7:00 PM) with ad libitum access to food and water. Before optogenetic stimulation, animals were lightly shaved from behind the ears to just past the shoulders. Testing was conducted in a custom behavioral chamber consisting of a 10 × 5 inch enclosure with three black matte acrylic walls and an optically clear front panel, with a clear acrylic center divider allowing visual contact between animals on either side. The floor was white matte acrylic covered with a disposable soft cloth surface that was changed between each cage to prevent olfactory contamination and maintain consistent testing conditions. The black matte walls minimize reflections; the optically clear front panel allows observation and recording during testing without obstruction. Prior to behavioral testing, animals underwent a structured habituation protocol ≥ 2 days. Each session consisted of 90 minutes of room habituation followed by 30 minutes of chamber habituation, after which animals were returned to their home cage.

Optogenetic stimulation, testing protocol, and behavioral scoring

Transcutaneous optogenetic stimulation was delivered using a LEDD1B LED driver with an M470F3 470nm LED coupled to an M41L01 patch cable (600 µm core diameter, 0.48 NA, Thorlabs). The fiber tip was held at a fixed distance of six inches from the animal's skin surface, producing a spot area of ~0.71 cm^2. Tip power was measured at each nominal setting using a calibrated power meter prior to each experiment. For mice, stimulation was delivered at powers of 1.5–3.5 mW (2.31–4.97 mW/cm^2). The 1.5 mW setting (2.31 mW/cm^2) served as a sub-threshold light-on control producing no behavioral response. For rats, stimulation was delivered at 4–12 mW (6.38–17.65 mW/cm^2). Irradiance values were calculated from measured tip power and a fixed 0.71 cm^2 spot area at 6-inch tip-to-animal distance.

For each session, mice and rats were allowed a brief settling period of around five minutes, then the LED fiber tip was positioned above the neck and shoulder region of the animal. Light was delivered in brief movements across the stimulation area while the experimenter monitored for wet dog shake (WDS) responses in real time. Sessions were recorded from front and top camera views using DMK 33UX287 cameras (The Imaging Source) at 120 frames per second in uncompressed format.

Videos were scored manually frame by frame using VirtualDub software. For each animal and stimulation trial, several parameters were recorded, including wet dog shake response (positive/negative), stimulus onset frame or time, event onset (head cock, full WDS, end) frame or time, latency to WDS onset (in seconds). A WDS was defined as a discrete bout of rapid oscillatory movements of the head and upper trunk, consistent with previously published criteria (*50*, *59*).

Oil/water stimulation (rats)

Animals were habituated in the acrylic chambers as above and settled for at least five minutes before stimulation. Once animals appeared at ease, room temperature water or sunflower seed oil (Sigma, S5007) was applied onto the upper back of the animals, targeting the region between the shoulder blades. Water and oil stimuli were applied on different days and thus during separate recording sessions.

Oil/water stimulation (naked mole-rats)

Naked mole-rats were habituated for five minutes in a 5 × 2 × 6 inch (length × width × height) acrylic arena. Following habituation, either mineral oil (Sigma-Aldrich, M8410-1L) or water was applied to the upper back/nape of the animal’s neck. Animals were then recorded for an additional five minutes at 240 frames per second. The number of WDSs, duration of scratching bouts, and duration of grooming bouts during the five-

bioRxiv preprint doi: https://doi.org/10.64898/2026.09.19.752904; this version posted September 21, 2026. The copyright holder for this preprint (which was not certified by peer review) is the author/funder, who has granted bioRxiv a license to display the preprint in perpetuity. It is made available under a CC-BY 4.0 International license.

minute post-application period were quantified from video recordings. Although naked mole-rats never performed WDSs following oil or water stimuli, each one always immediately attended to the oil and water stimuli right after stimulus presentation, often by orienting their head in the direction from which the stimulus was applied, indicating that they sensed the oil or water applied. Naked mole-rats spent most of each behavioral recording session displaying typical locomotion, burrowing, and digging behaviors.

In Vivo Epifluorescence Ca^{2+} Imaging

Mice of both sexes at 4 – 6 weeks were anesthetized with inhalational isoflurane (Covetrus, 29405) throughout the experiments. Back hair was shaved, back skin disinfected, leg hair removed, and an incision was made over the lumbar vertebrae. Paravertebral muscles along the L4 vertebral spine were dissected, then the bone on top of the L4 DRG was removed by rongeur. A spinal clamp was used to stabilize the spinal column. The surgical preparation was then placed under an upright epifluorescence microscope (Zeiss Axio Examiner) with a 10X air objective (Zeiss Epiplan, NA=0.20). The light source was a 470nm LED (M470L5, Thorlabs) with an LED driver (LEDD1B, Thorlabs). A CMOS Camera (CS505MU1, Thorlabs) was triggered at five or ten frames per second with a 50 ms exposure time. All the recorded stimuli were synchronized with the camera and LED using a DAQ board (National Instrument, NI USB-6343).

Stimuli

Chemical stimuli were prepared by dissolving in 0.9% saline (0.9% Saline Solution, Teknova, S5825) shortly before application. Stock capsaicin (Sigma-Aldrich, M2028-250MG) was dissolved in DMSO at 10% weight/volume. Stock capsaicin and AITC (allyl isothiocyanate, Sigma, 377430-500G) were diluted in 0.9% saline and applied onto the DRG in succession from 1:10,000, then 1:1,000, then 1:100, then 1:10, until Ca^{2+} responses were observed. Given the volatility of AITC and imperfect solubility of capsaicin in saline, we predicted that different concentrations could elicit Ca^{2+} responses across trials, even within the same animal (e.g., between the left and right L4 DRGs). In the case of all mouse DRGs, responses to capsaicin were not observed at any concentrations, including at 1:10, while responses to AITC were readily observed at concentrations usually orders of magnitude lower. In the case of rat DRGs, clear responses to both AITC and capsaicin were typically easily observed at concentrations orders of magnitude lower than 1:10. The first concentration at which responses were observed constituted the last application of that chemical stimulus to avoid over-stimulating DRG neurons and triggering cytotoxicity. Between application of different chemical stimuli, three washes of 20 µL saline were applied, and DRGs were allowed to recover for ten minutes with no stimuli. Cotton swabs were used to gently remove excess chemical or saline stimuli between trials or during washes.

All DRGs were stimulated with a standard sequence: mechanical stimulation (leg pinch) first, followed by saline (intended as a chemical negative control), followed by AITC, then capsaicin. AITC and capsaicin application order was reversed in some animals, with no obvious differences. After all chemical stimulations were concluded, a final round of mechanical (pinch) stimulation was often performed to confirm the health of the DRG neurons at the end of the trial. During all trials, stimuli were not applied during frames 1 – 100, allowing these frames to constitute a baseline period for each stimulus trial. During mechanical stimulation, forceps were used to pinch the hairy hindlimb during frames 101 – 300 and the hindpaw during frames 301 – 500. Videos were captured at 10 Hz during mechanical stimulus trials and 5 Hz during chemical stimulus trials. All chemical (including negative control saline) stimuli were applied onto the DRG between frames 101 and 150 and liquid was removed from the DRG by frame 200. Chemical responses were quantified starting from frame 201. Mechanical stimulus trials lasted 60 seconds, with the last 10 seconds not having any stimuli; mechanical stimulus trials thus yielded 500 frames in total. Chemical stimulus trials lasted 120 seconds, with the second 20 seconds (frames 101 – 200) being spliced out due to chemical application and removal; chemical stimulus trials thus also yielded 500 frames in total.

Calcium imaging processing and analysis

For imaging analysis, initial processing was conducted in Fiji (ImageJ; NIH, https://imagej.net/Fiji/Downloads). Motion correction and spatial high-pass filtering was conducted using a

bioRxiv preprint doi: https://doi.org/10.64898/2026.09.19.752904; this version posted September 21, 2026. The copyright holder for this preprint (which was not certified by peer review) is the author/funder, who has granted bioRxiv a license to display the preprint in perpetuity. It is made available under a CC-BY 4.0 International license.

custom-written macro utilizing ImageJ plugins moco and Unsharp Mask. Regions of interest corresponding to cells that exhibited baseline signal and/or calcium response to any stimuli were selected, their coordinates aligned across videos, and their mean fluorescence intensity measurements recorded from across all stimulus trials.

The mean fluorescence intensity measurements from Fiji were further processed and analyzed using MATLAB. To calculate ΔF/F, F was defined for each trial as the minimum of the mean or median of the fluorescence intensity during the baseline 100 frame period before each stimulus. ΔF/F measurements were then normalized for plotting in heatmaps. For the heatmaps in Figs. 5 and fig. S13, data were filtered to include only cells that responded to at least one chemical and then ordered by responses to AITC then to capsaicin. For the heatmap in Fig. 6, data were filtered to include only cells that responded to at least one chemical and to mechanical stimulation, grouped by responsiveness to mechanical stimulation in the 100 – 300 frame range (the hairy hindlimb mechanical stimulation period), then the 300 – 500 frame range (the hindpaw mechanical stimulation period), and then ordered by responses to AITC then to capsaicin.

bioRxiv preprint doi: https://doi.org/10.64898/2026.09.19.752904; this version posted September 21, 2026. The copyright holder for this preprint (which was not certified by peer review) is the author/funder, who has granted bioRxiv a license to display the preprint in perpetuity. It is made available under a CC-BY 4.0 International license.

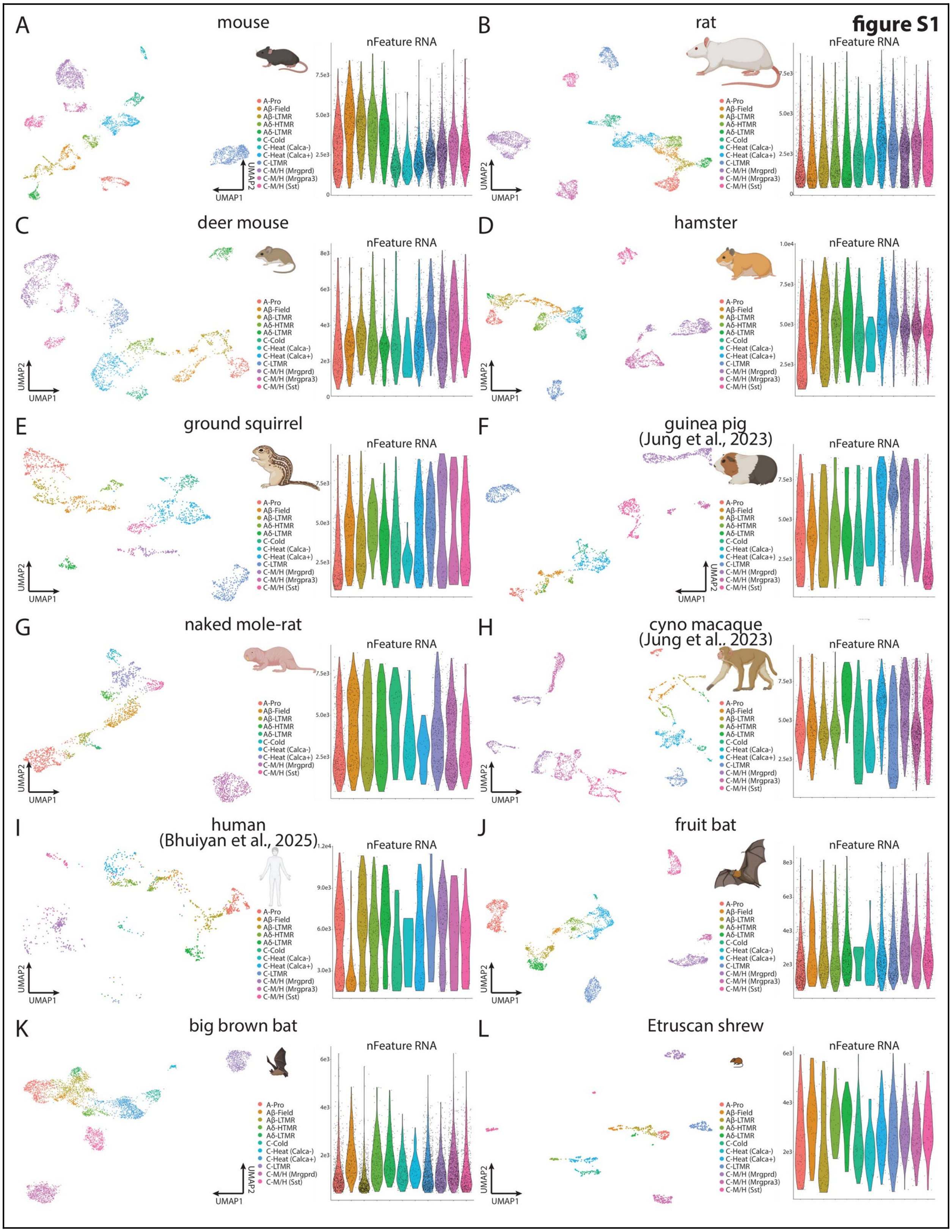

Fig. S1. Example DRG neuron single-nucleus RNA-sequencing replicate datasets.

(**A** to **L**) Left, Unform Manifold Approximation and Projection (UMAP) plots of DRG neuron single-nucleus RNA-sequencing datasets. Datasets plotted are from single biological replicates from distinct individual animals for all

bioRxiv preprint doi: https://doi.org/10.64898/2026.09.19.752904; this version posted September 21, 2026. The copyright holder for this preprint (which was not certified by peer review) is the author/funder, who has granted bioRxiv a license to display the preprint in perpetuity. It is made available under a CC-BY 4.0 International license.

species except guinea pig (F), naked mole-rat (G), cynomolgous macaque (H), and human (I). Guinea pig and cynomolgous macaque datasets are from (*33*) and the human dataset is from (*35*). UMAP datapoints are colored by DRG neuron orthotype. Right, quality control nFeature RNA (genes per cell) metric plotted for each orthotype of each replicate for each species.

bioRxiv preprint doi: https://doi.org/10.64898/2026.09.19.752904; this version posted September 21, 2026. The copyright holder for this preprint (which was not certified by peer review) is the author/funder, who has granted bioRxiv a license to display the preprint in perpetuity. It is made available under a CC-BY 4.0 International license.

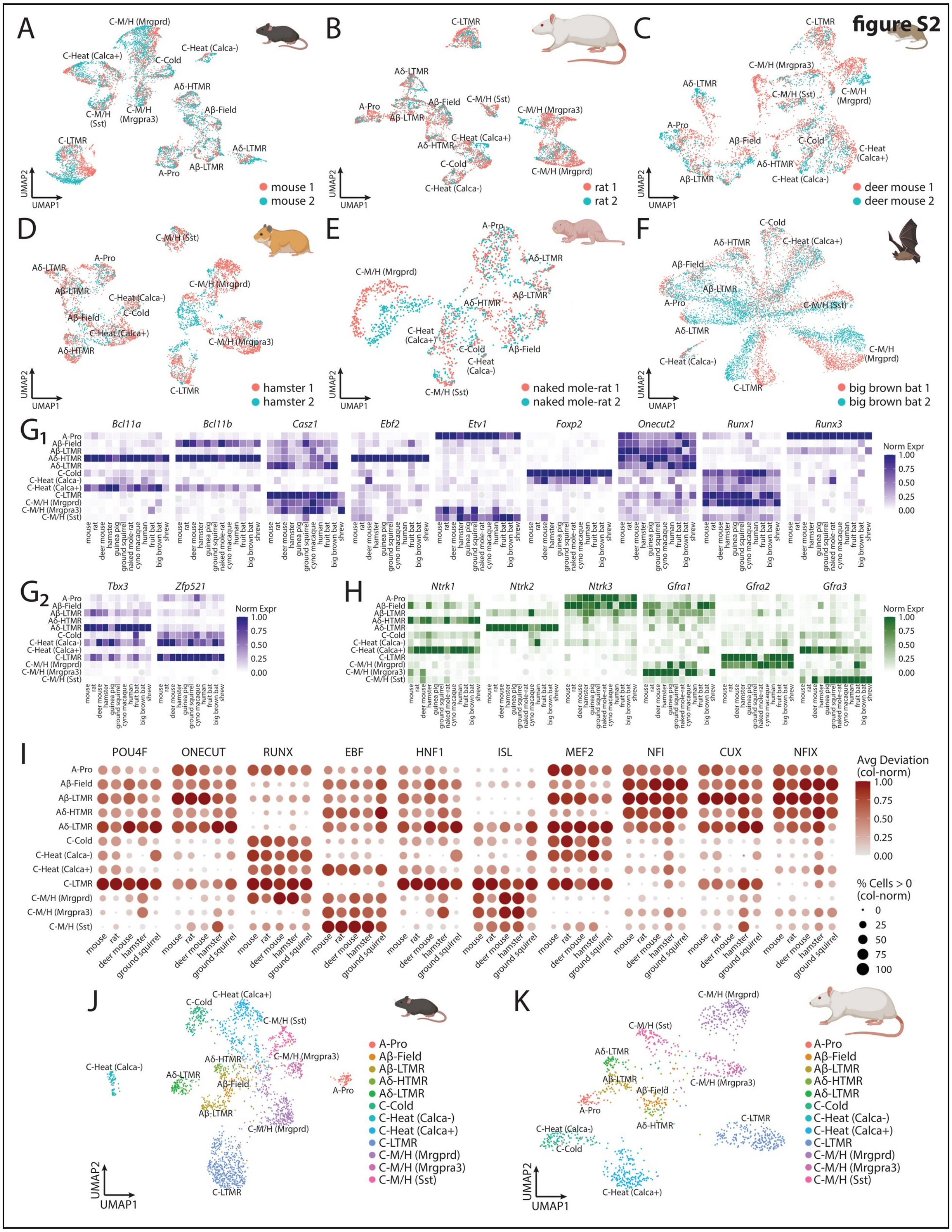

Fig. S2. Additional evidence of DRG neuron-type conservation across species from single-nucleus RNA- and ATAC-sequencing datasets.

bioRxiv preprint doi: https://doi.org/10.64898/2026.09.19.752904; this version posted September 21, 2026. The copyright holder for this preprint (which was not certified by peer review) is the author/funder, who has granted bioRxiv a license to display the preprint in perpetuity. It is made available under a CC-BY 4.0 International license.

(**A** to **F**) Comparison of biological replicates from different individual animals via UMAP plots of gene expression (RNA) datasets following within-species dataset merging (Seurat *merge* function), rather than integration, to preserve and display, rather than hide, any batch effects and technical variation. UMAP plot datapoints are colored by replicates (blue, red) and DRG neuron orthotypes are labeled on each plot. Sample species include mouse (A), rat (B), deer mouse (C), hamster (D), naked mole-rat (E), and big brown bat (F). Overlap between distinct replicates within clusters suggests biological similarity and high and consistent data quality across replicates.

(**G** and **H**) Orthotype-specific expression patterns of transcription factor (G, purple) and growth factor receptor (H, green) genes across species. Gray circles, missing cell types (see Fig. 2 and fig. S3).

(**I** and **J**) UMAP plots of ATAC datasets from example mouse (I) and rat (J) multiome datasets. Datapoints are colored by cell type.

bioRxiv preprint doi: https://doi.org/10.64898/2026.09.19.752904; this version posted September 21, 2026. The copyright holder for this preprint (which was not certified by peer review) is the author/funder, who has granted bioRxiv a license to display the preprint in perpetuity. It is made available under a CC-BY 4.0 International license.

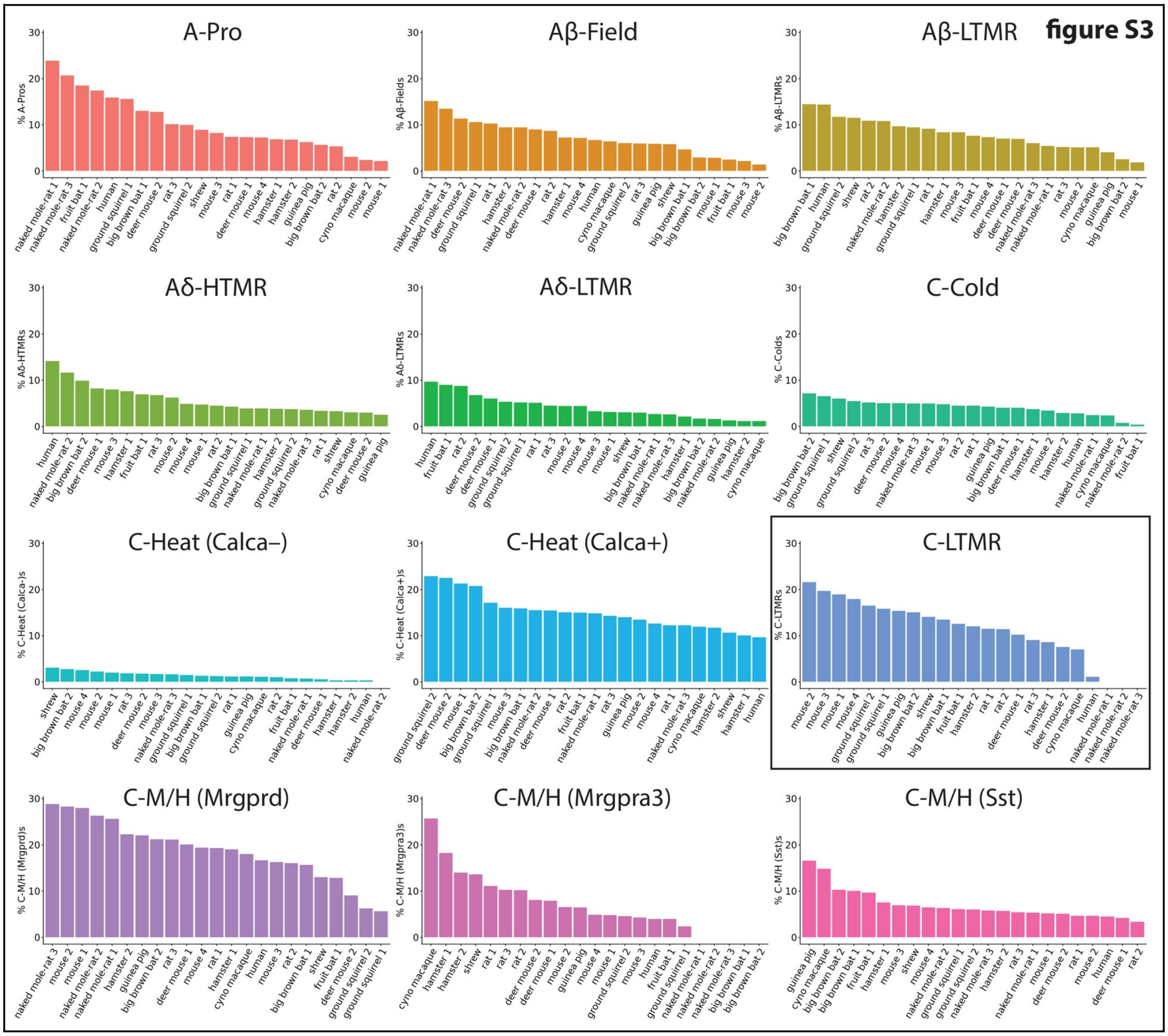

Fig. S3. DRG neuron orthotype proportions of individual samples across species.

DRG neuron orthotype proportions of individual sample replicates across species. Individual replicates are ordered by cell-type proportion within that replicate.

bioRxiv preprint doi: https://doi.org/10.64898/2026.09.19.752904; this version posted September 21, 2026. The copyright holder for this preprint (which was not certified by peer review) is the author/funder, who has granted bioRxiv a license to display the preprint in perpetuity. It is made available under a CC-BY 4.0 International license.

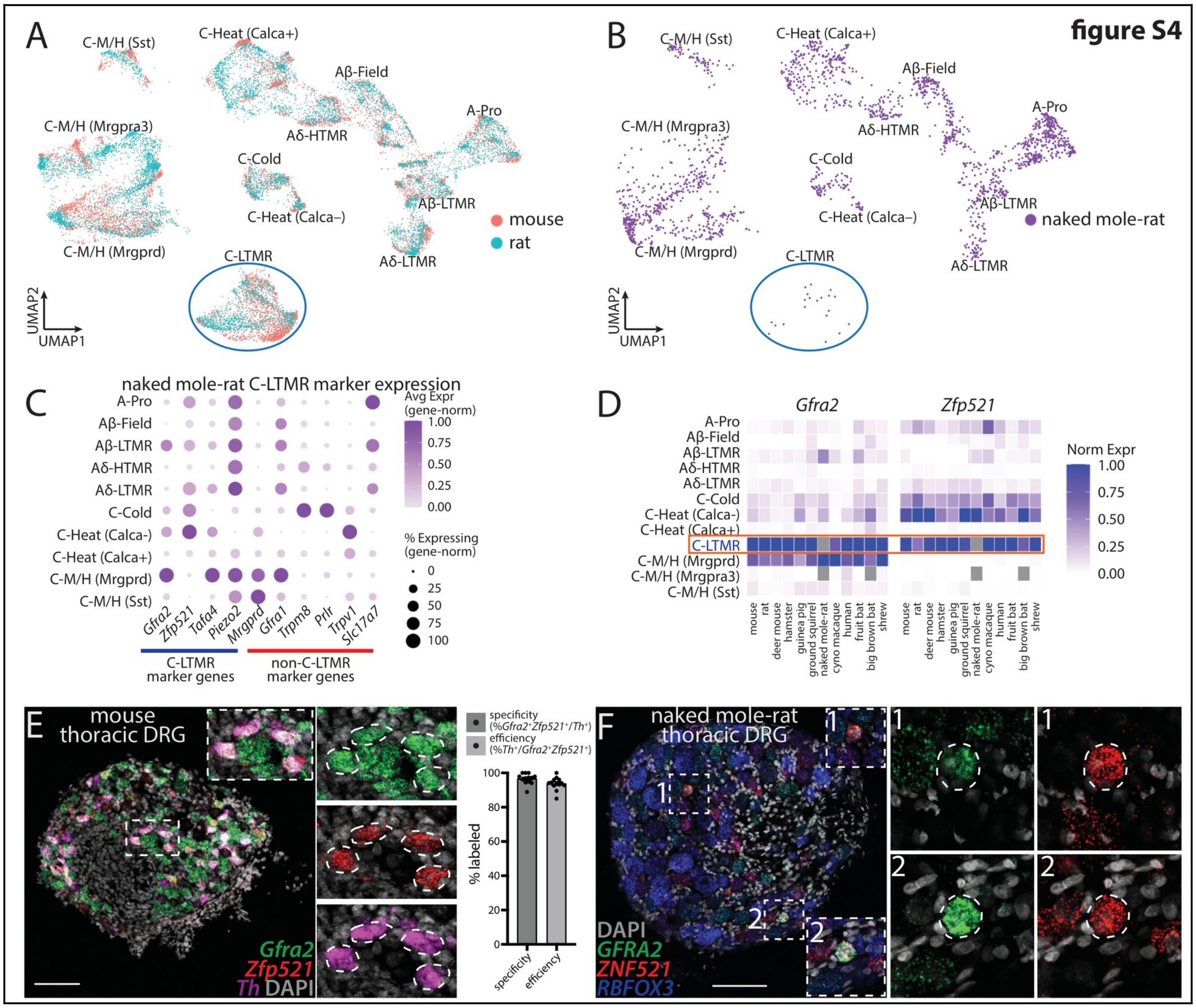

Fig. S4. Additional characterization of C-LTMR neuron proportions across species.

(**A**) Mouse (red) and rat (blue) samples in the pan-species UMAP embedding (see Fig. 1B). Note abundance of neurons from both mouse and rat in the C-LTMR cluster (circled in blue).

(**B**) Naked mole-rat samples in the pan-species UMAP embedding (see Fig. 1B). Note relative paucity of neurons in the C-LTMR cluster (circled in blue).

(**C**) Dot plot of C-LTMR and other DRG neuron orthotype marker genes in naked mole-rat samples.

(**D**) *Gfra2*/*GFRA2* and *Zfp521*/*ZNF521* expression patterns across DRG neuron orthotypes and species shows that these two genes are conserved co-markers of C-LTMRs (orange box) across species. Gray boxes, missing cell types.

(**E**) *In situ* hybridizations of mouse mid-thoracic DRGs showing overlap between *Gfra2* (green) and *Zfp521* (red), co-markers of C-LTMRs, and *Th* (magenta), a canonical marker of C-LTMRs in mice (*24*). Left, confocal images of *in situ* hybridizations. Right, quantifications; mean ± SEM.

(**F**) *In situ* hybridization of naked mole-rat mid-thoracic DRGs against *GFRA2* (green) and *ZNF521* (red), co-markers of C-LTMRs, along with pan-neuronal marker *Rbfox3*/*RBFOX3* (NeuN, blue), sometimes reveal a small number (e.g., 1 – 5) of *GFRA2*$^{+}$/*ZNF521*$^{+}$ putative C-LTMRs per DRG, as outlined in dotted white lines in insets (right).

Scale bars, 100 µm.

bioRxiv preprint doi: https://doi.org/10.64898/2026.09.19.752904; this version posted September 21, 2026. The copyright holder for this preprint (which was not certified by peer review) is the author/funder, who has granted bioRxiv a license to display the preprint in perpetuity. It is made available under a CC-BY 4.0 International license.

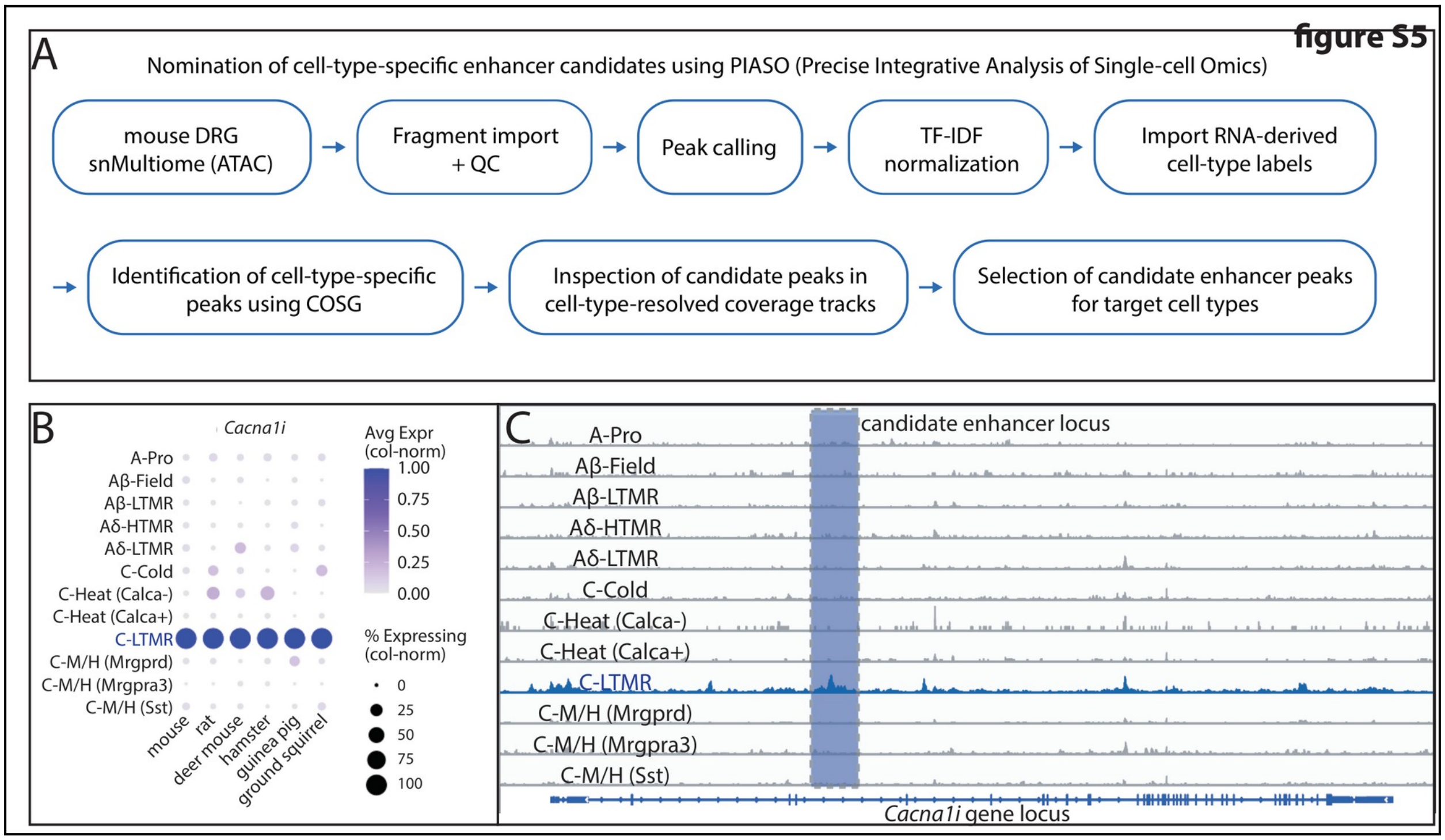

Fig. S5. Enhancer candidate selection pipeline for C-LTMR neuron targeting.

(**A**) Schematic illustrating PIASO pipeline for candidate enhancer selection. ATAC fragments were imported, with quality control per nucleus by fragment count and TSS enrichment; accessible peaks were then called. A cell × peak matrix was assembled and normalized by TF-IDF. Cell-type annotations transferred from the paired snRNA-seq analysis (matched by cell barcode) were used to group nuclei, and cell-type-specific marker peaks were identified with COSG. Candidate peaks were then inspected in cell-type-resolved coverage tracks, and enhancer candidates for each target cell type were selected from the regions surrounding peak summits. Vertebrate phastCons conservation was used to further prioritize candidates. TF-IDF, term frequency–inverse document frequency.

(**B**) Dot plot showing specific expression of C-LTMR marker gene *Cacna1i* across rodent species.

(**C**) Genome tracks showing enrichment of ATAC signal in top C-LTMR candidate *cis*-regulatory element in an intron of C-LTMR marker gene *Cacna1i* relative to other DRG neuron types. Blue shaded region corresponds to selected candidate sequence. See also Fig. 3A.

bioRxiv preprint doi: https://doi.org/10.64898/2026.09.19.752904; this version posted September 21, 2026. The copyright holder for this preprint (which was not certified by peer review) is the author/funder, who has granted bioRxiv a license to display the preprint in perpetuity. It is made available under a CC-BY 4.0 International license.

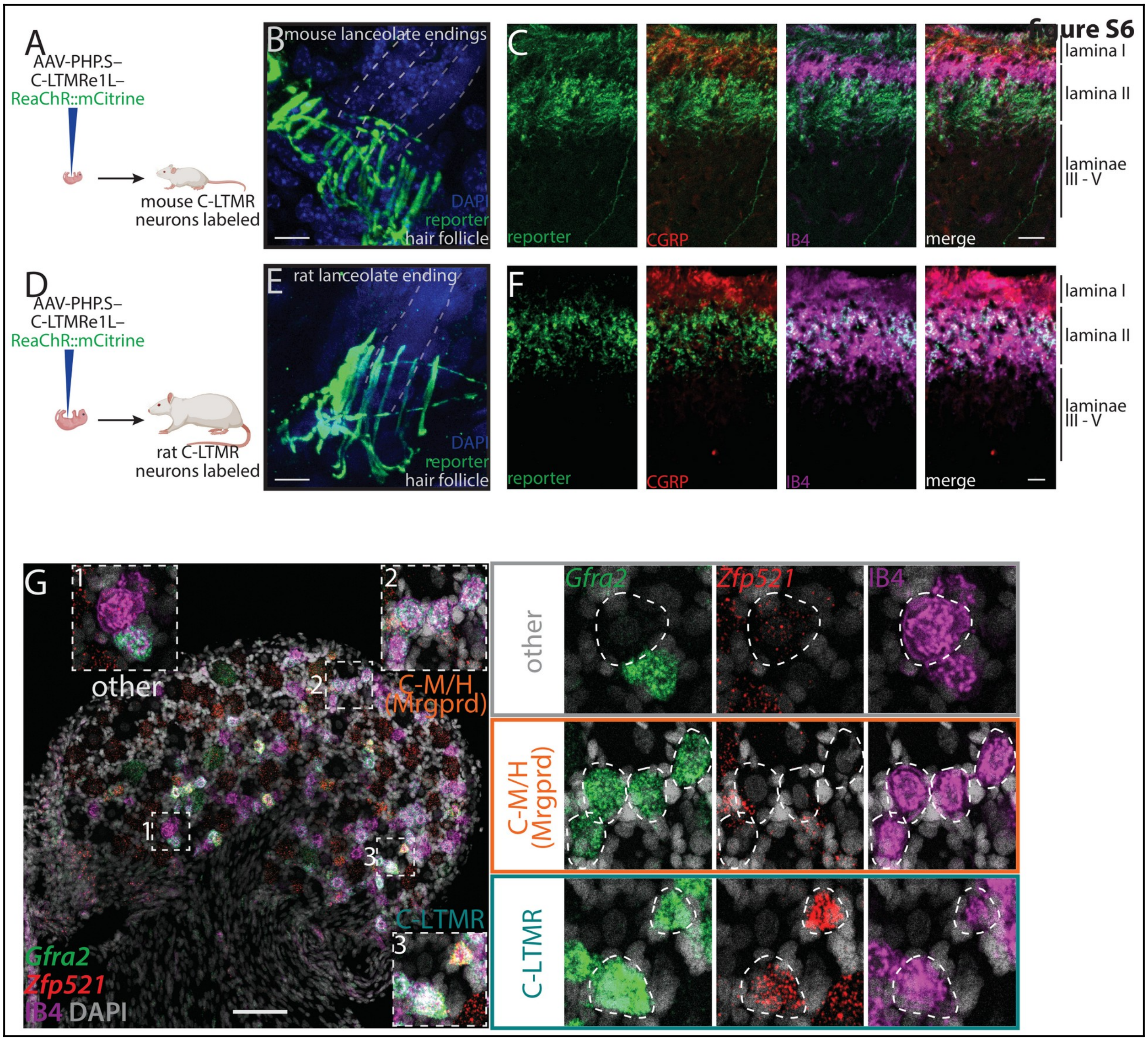

Fig. S6. eAAV-mediated characterization of C-LTMR peripheral and central anatomy across mice and rats.

(**A** to **F**) Schematics of eAAV-mediated C-LTMR targeting in mice (A) and rats (D). Labeling of lanceolate hair follicle endings in hairy skin of mice (B) and rats (E). eAAV-mediated C-LTMR neuron labeling in mice (C) and rats (F) reveals central terminals targeting spinal cord dorsal horn lamina II, ventral to lamina I marker CGRP (see also fig. S3D). Scale bars, 20 µm.

(**G**) *In situ* hybridization characterization of IB4-binding rat DRG neurons. Rat IB4-binding neurons are a more diverse population than in mice. In mice, almost all (96%) IB4-binding neurons express *Mrgprd*, a marker of mouse C-M/H (Mrgprd) neurons (see fig. S12, B and C), while in rats, IB4-binding neurons (right insets) include *Gfra2*$^{+}$/*Zfp521*$^{-}$ putative C-M/H (Mrgprd) neurons, as well as *Gfra2*$^{+}$/*Zfp521*$^{+}$ putative C-LTMR neurons and other *Gfra2*$^{-}$ neurons. Thus, IB4 binding may not mark the same DRG neurons and spinal laminae and sublaminae in rats as in mice (*118*, *119*). Scale bar, 100 µm.

bioRxiv preprint doi: https://doi.org/10.64898/2026.09.19.752904; this version posted September 21, 2026. The copyright holder for this preprint (which was not certified by peer review) is the author/funder, who has granted bioRxiv a license to display the preprint in perpetuity. It is made available under a CC-BY 4.0 International license.

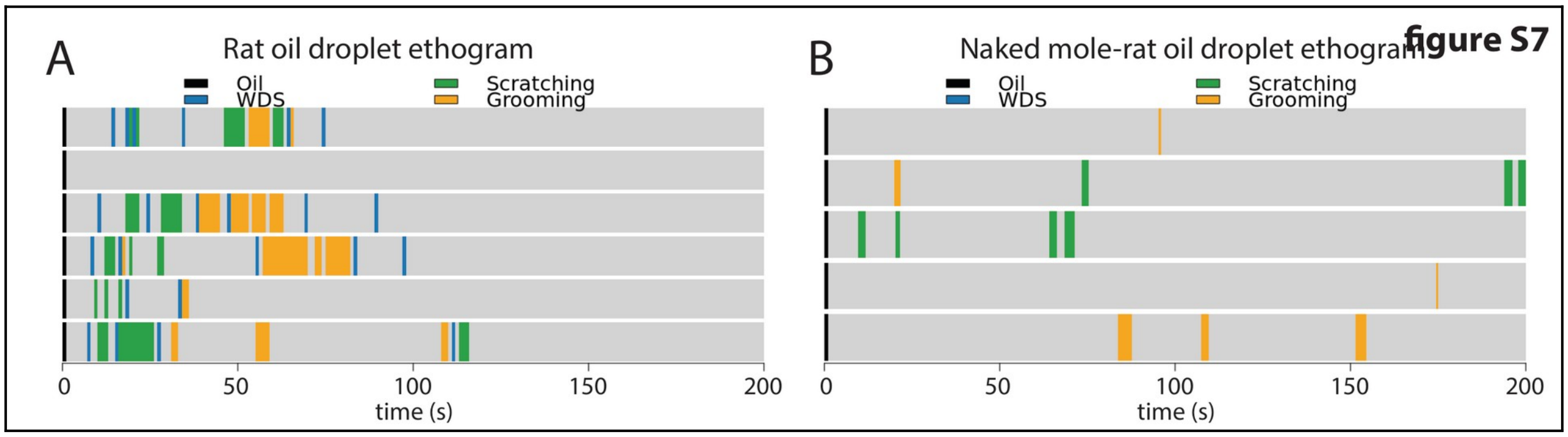

Fig. S7. Characterization of rat and naked mole-rat somatomotor behavioral responses to oil application on the upper back.

(**A** and **B**) Ethograms of rat (A) and naked mole-rat (B) responses to oil droplets targeted to the upper back. WDS, wet-dog shake. Black, blue, green, and yellow bars correspond to oil stimulus presentation, WDS bouts, bouts of scratching using the hindlimb, and bouts of facial and/or upper trunk grooming, respectively. Rats frequently perform WDS following oil stimulus presentation and other grooming/scratching behaviors, while naked mole-rats never perform WDS but do occasionally engage in other scratching and grooming behaviors.

bioRxiv preprint doi: https://doi.org/10.64898/2026.09.19.752904; this version posted September 21, 2026. The copyright holder for this preprint (which was not certified by peer review) is the author/funder, who has granted bioRxiv a license to display the preprint in perpetuity. It is made available under a CC-BY 4.0 International license.

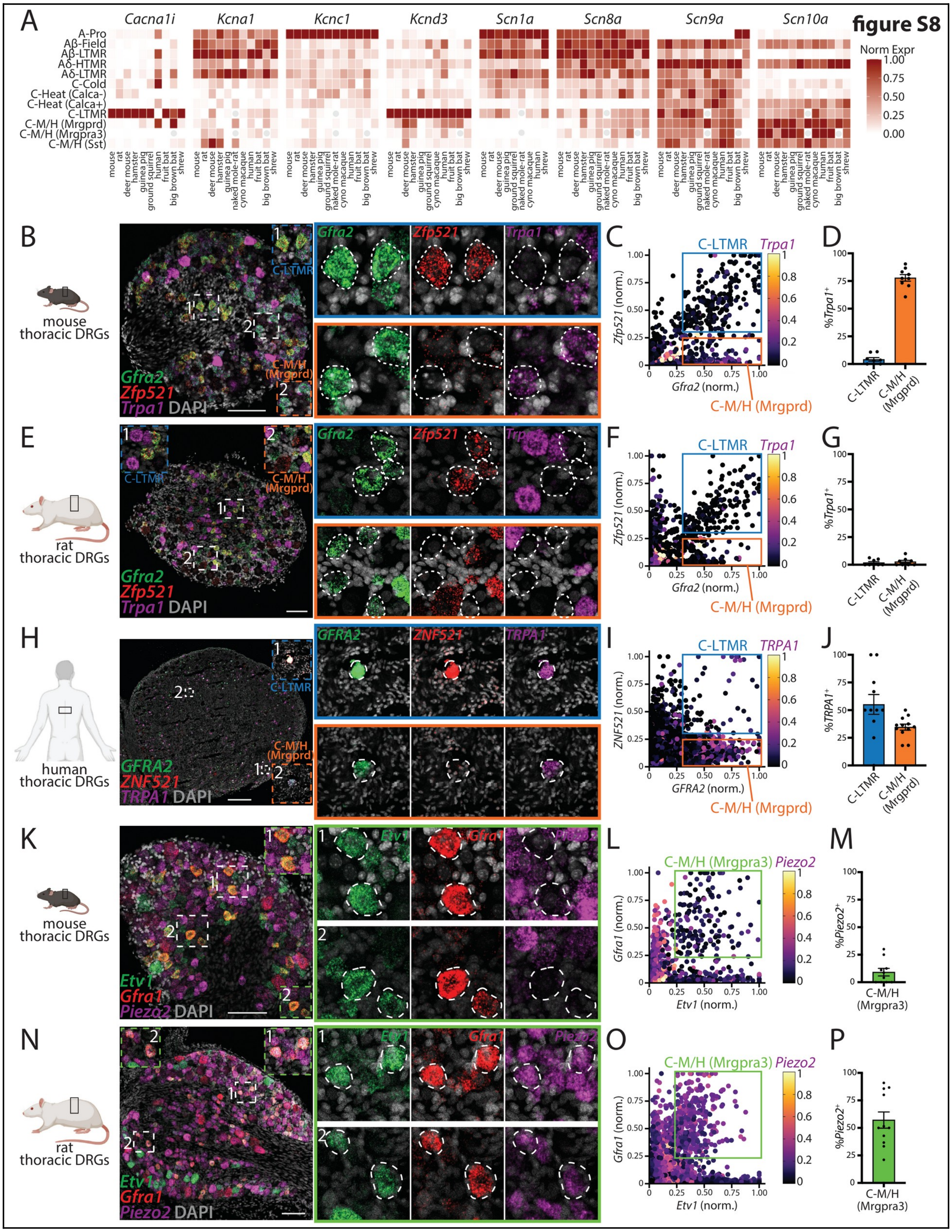

Fig. S8. Additional validation of within-orthotype sensor shuffling across species.

(**A**) Gene expression plot of orthotype ion channel expression across species. Gray circles, missing cell types (see Fig. 2 and fig. S3).

bioRxiv preprint doi: https://doi.org/10.64898/2026.09.19.752904; this version posted September 21, 2026. The copyright holder for this preprint (which was not certified by peer review) is the author/funder, who has granted bioRxiv a license to display the preprint in perpetuity. It is made available under a CC-BY 4.0 International license.

(**B** to **J**) *In situ* hybridization validation of *Trpa1* expression patterns in C-LTMR (*Gfra2*$^{+}$/*Zfp521*$^{+}$) and C-M/H (Mrgprd) (*Gfra2*$^{+}$/*Zfp521*$^{-}$) neurons across mouse (B), rat (E), and human (H) mid-thoracic DRGs. (C), (F), and (I): Quantification of neuronal *Trpa1* expression (1001, 1540, and 4367 neurons from 9, 9, and 12 sections from 3, 4, and 4 DRGs from 2, 3, and 2 mice, rats, and humans, respectively). (D), (G), and (J): Percentages of *Trpa1*$^{+}$ neurons (9, 9, and 12 DRG sections from mice, rats, and humans, respectively); mean ± SEM.

(**K** to **P**) *In situ* hybridization validation of *Piezo2* expression patterns in C-M/H (Mrgpra3) (*Etv1*$^{+}$/*Gfra1*$^{+}$) neurons across mouse (K) and rat (N) mid-thoracic DRGs. (L) and (O): Quantification of neuronal *Piezo2* expression (1294 and 3070 neurons from 10 and 11 sections from 5 and 5 DRGs from 3 and 3 mice and rats, respectively). (M) and (P): Percentages of *Piezo2*$^{+}$ neurons (10 and 11 DRG sections from mice and rats, respectively); mean ± SEM.

Scale bars, 100 µm (B, E, K, N), 500 µm (H).

bioRxiv preprint doi: https://doi.org/10.64898/2026.09.19.752904; this version posted September 21, 2026. The copyright holder for this preprint (which was not certified by peer review) is the author/funder, who has granted bioRxiv a license to display the preprint in perpetuity. It is made available under a CC-BY 4.0 International license.

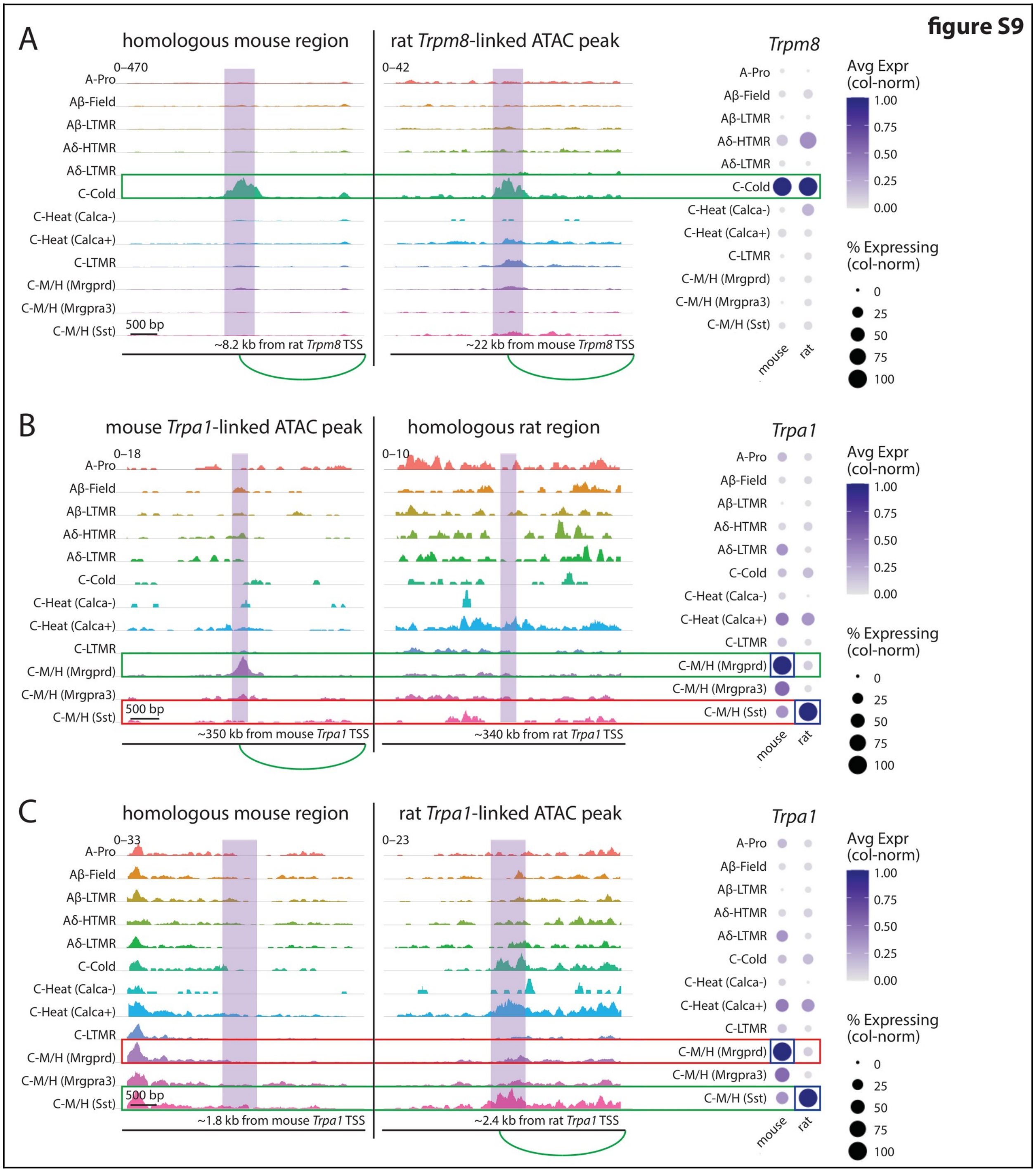

Fig. S9. Species-specific utilization of genomic elements that may regulate sensor expression.

(**A**) Left and middle, genome tracks showing ATAC signal across DRG neuron orthotypes in mouse (left) and rat (middle) genomic coordinates surrounding a rat *Trpm8*-linked ATAC peak (see Methods) and the corresponding homologous mouse genomic region. Right, dot plot showing *Trpm8* expression in mouse and rat DRG neuron orthotypes. C-Cold neurons, which express high *Trpm8* in both species, exhibit large, clear peaks near *Trpm8* in both rats and mice.

(**B**) Left and middle, genome tracks showing ATAC signal across DRG neuron orthotypes in mouse (left) and rat (middle) genomic coordinates surrounding a mouse *Trpa1*-linked ATAC peak (see Methods) and the corresponding

bioRxiv preprint doi: https://doi.org/10.64898/2026.09.19.752904; this version posted September 21, 2026. The copyright holder for this preprint (which was not certified by peer review) is the author/funder, who has granted bioRxiv a license to display the preprint in perpetuity. It is made available under a CC-BY 4.0 International license.

homologous rat genomic region. Right, dot plot showing *Trpa1* expression in mouse and rat DRG neuron orthotypes. Note that C-M/H (Mrgprd) neurons have the highest *Trpa1* expression in mice, while C-M/H (Sst) neurons have the highest *Trpa1* expression in rats. While mouse C-M/H (Mrgprd) neurons exhibit a clear peak linked to *Trpa1* expression, neither rat C-M/H (Mrgprd) neurons, which do not express appreciable *Trpa1*, nor rat C-M/H (Sst) neurons, which do express *Trpa1*, exhibit an ATAC peak at the homologous location.

(**C**) Left and middle, genome tracks showing ATAC signal across DRG neuron orthotypes in mouse (left) and rat (middle) genomic coordinates surrounding a rat *Trpa1*-linked ATAC peak (see Methods) and the corresponding homologous mouse genomic region. Right, dot plot showing *Trpa1* expression in mouse and rat DRG neuron orthotypes. Note that C-M/H (Sst) neurons have the highest *Trpa1* expression in rats, while C-M/H (Mrgprd) neurons have the highest *Trpa1* expression in mice. While rat C-M/H (Sst) neurons exhibit a clear peak linked to *Trpa1* expression, neither mouse C-M/H (Mrgprd) nor C-M/H (Sst) neurons exhibit an ATAC peak at the homologous location.

bioRxiv preprint doi: https://doi.org/10.64898/2026.09.19.752904; this version posted September 21, 2026. The copyright holder for this preprint (which was not certified by peer review) is the author/funder, who has granted bioRxiv a license to display the preprint in perpetuity. It is made available under a CC-BY 4.0 International license.

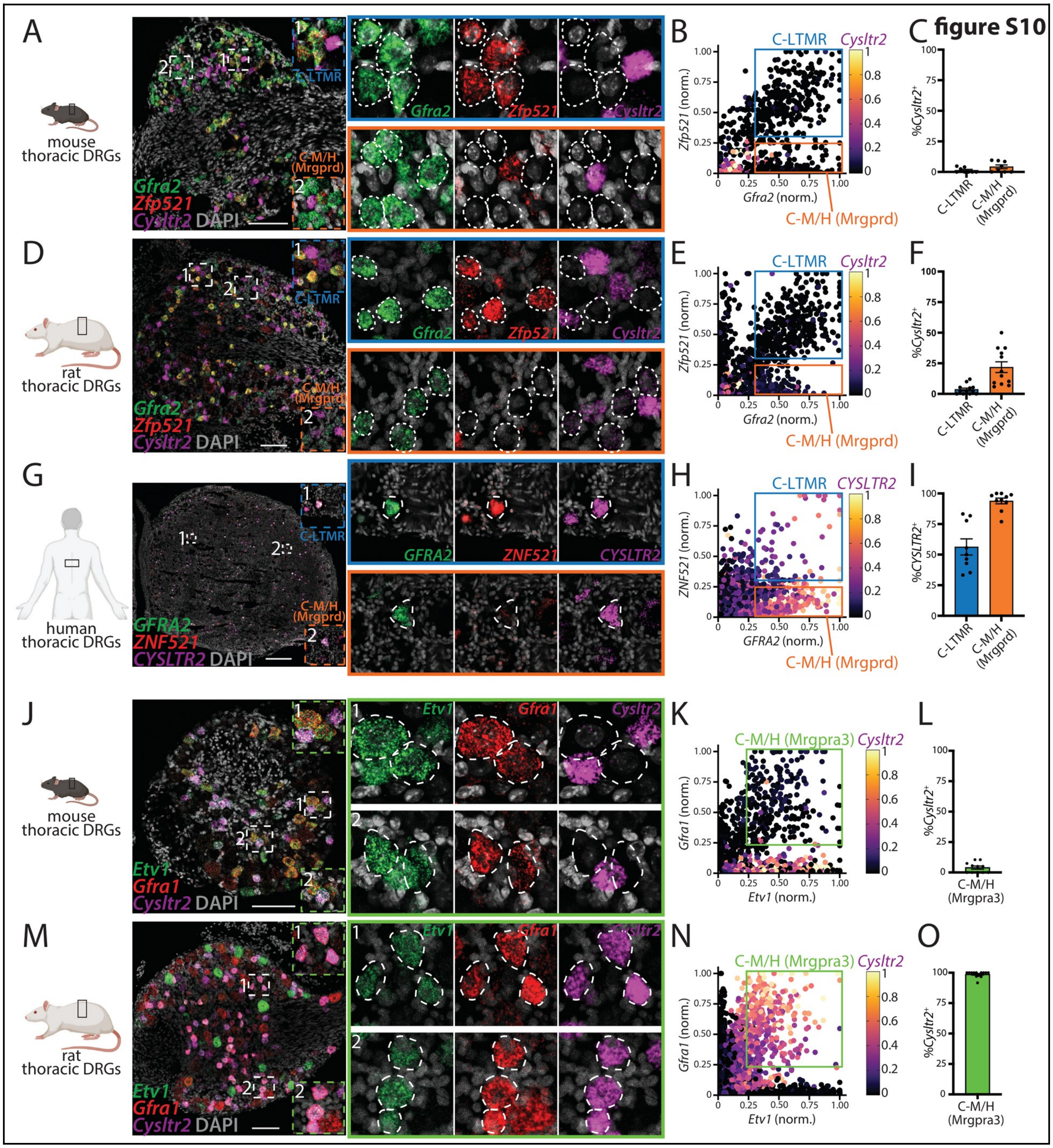

Fig. S10. Validation of immune receptor shuffling across species.

(**A** to **I**) *In situ* hybridization validation of *Cysltr2* expression patterns in C-LTMR (*Gfra2*$^{+}$/*Zfp521*$^{+}$) and C-M/H (Mrgprd) (*Gfra2*$^{+}$/*Zfp521*$^{-}$) neurons across mouse (A), rat (D), and human (G) mid-thoracic DRGs. (B), (E), and (H): Quantification of neuronal *Cysltr2* expression (1132, 2468, and 3430 neurons from 8, 12, and 10 sections from 4, 4, and 4 DRGs from 2, 2, and 2 mice, rats, and humans, respectively). (C), (F), and (I): Percentages of *Cysltr2*$^{+}$ neurons (8, 12, and 10 DRG sections from mice, rats, and humans, respectively); mean ± SEM.

(**J** to **O**) *In situ* hybridization validation of *Cysltr2* expression patterns in C-M/H (Mrgpra3) (*Etv1*$^{+}$/*Gfra1*$^{+}$) neurons across mouse (J) and rat (M) mid-thoracic DRGs. (K) and (N): Quantification of neuronal *Cysltr2* expression (1147

bioRxiv preprint doi: https://doi.org/10.64898/2026.09.19.752904; this version posted September 21, 2026. The copyright holder for this preprint (which was not certified by peer review) is the author/funder, who has granted bioRxiv a license to display the preprint in perpetuity. It is made available under a CC-BY 4.0 International license.

and 2488 neurons from 14 and 13 sections from 6 and 5 DRGs from 3 and 3 mice and rats, respectively). (L) and (O): Percentages of *Cysltr2*$^{+}$ neurons (14 and 13 DRG sections from mice and rats, respectively); mean ± SEM. Scale bars, 100 μm (A, D, J, M), 500 μm (G).

bioRxiv preprint doi: https://doi.org/10.64898/2026.09.19.752904; this version posted September 21, 2026. The copyright holder for this preprint (which was not certified by peer review) is the author/funder, who has granted bioRxiv a license to display the preprint in perpetuity. It is made available under a CC-BY 4.0 International license.

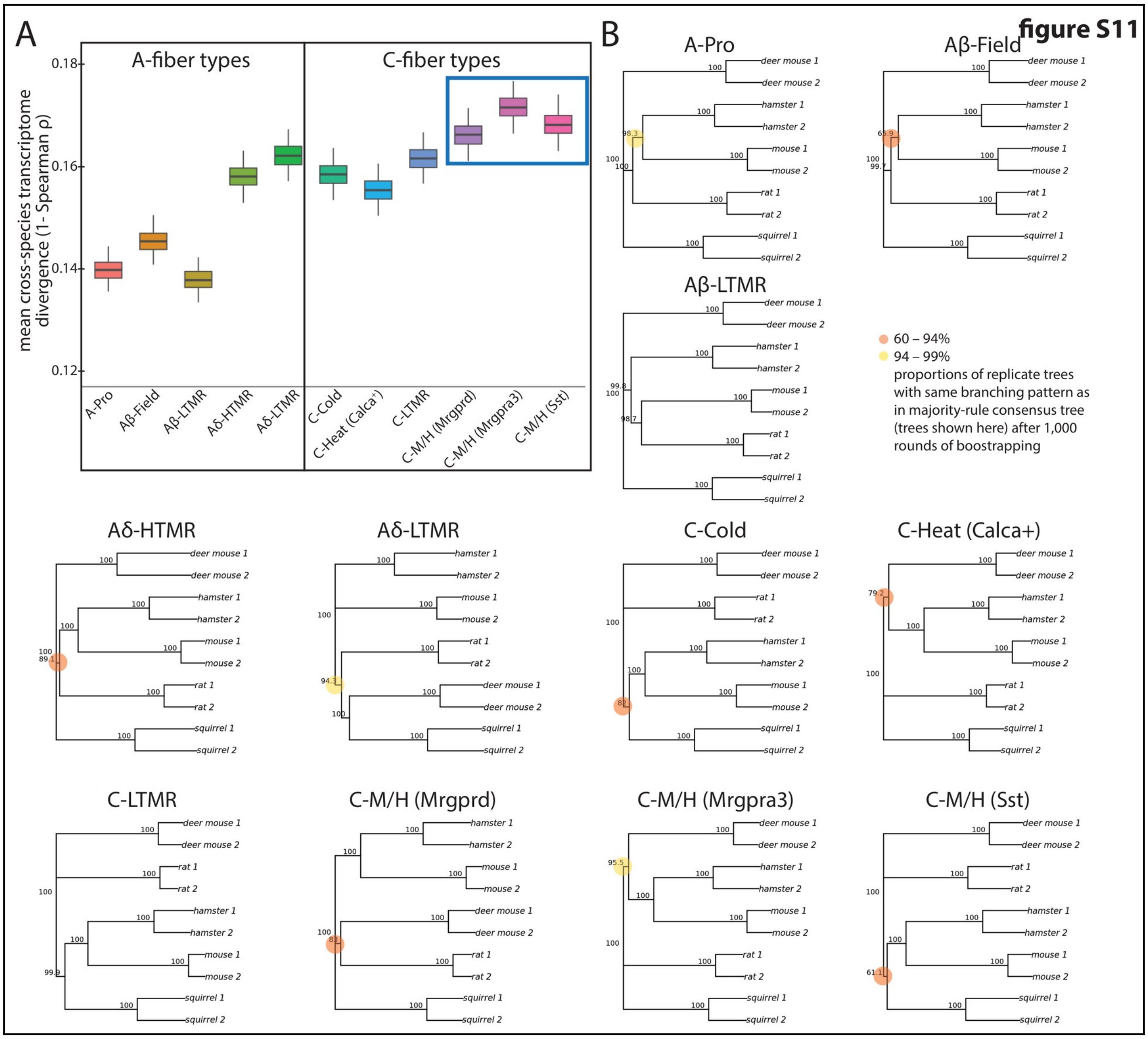

Fig. S11. Characterization of DRG neuron orthotype transcriptomic divergence across mammals.

(**A**) Total branch lengths of rodent DRG neuron orthotype gene expression trees (sum of transcriptomic distance/divergence values; see Methods). Box plots show the median (central value), upper and lower quartile (box limits), and 95% confidence intervals (whiskers) for 1,000 bootstrap replicates. Blue box highlights the three polymodal C-fiber (C-M/H) neuron orthotypes with the highest transcriptomic divergence values.

(**B**) Rodent DRG neuron orthotype gene expression phylogenetic trees (see Methods). Neighbor-joining trees are generated using pairwise gene expression distances (1− ρ, Spearman's correlation coefficient) between orthotypes both within and across species. Tree sizes shown here are normalized within orthotype. Bootstrap values (proportions of replicate trees with the same branching pattern as in the majority-rule consensus tree shown) lower than 99% are indicated by colored circles at the corresponding nodes. Orthotype gene expression phylogenetic trees largely do not recapitulate known phylogenetic relationships between species, but the invariability of intra-species replicate pairing demonstrates consistent gene expression patterns within orthotype across species.

bioRxiv preprint doi: https://doi.org/10.64898/2026.09.19.752904; this version posted September 21, 2026. The copyright holder for this preprint (which was not certified by peer review) is the author/funder, who has granted bioRxiv a license to display the preprint in perpetuity. It is made available under a CC-BY 4.0 International license.

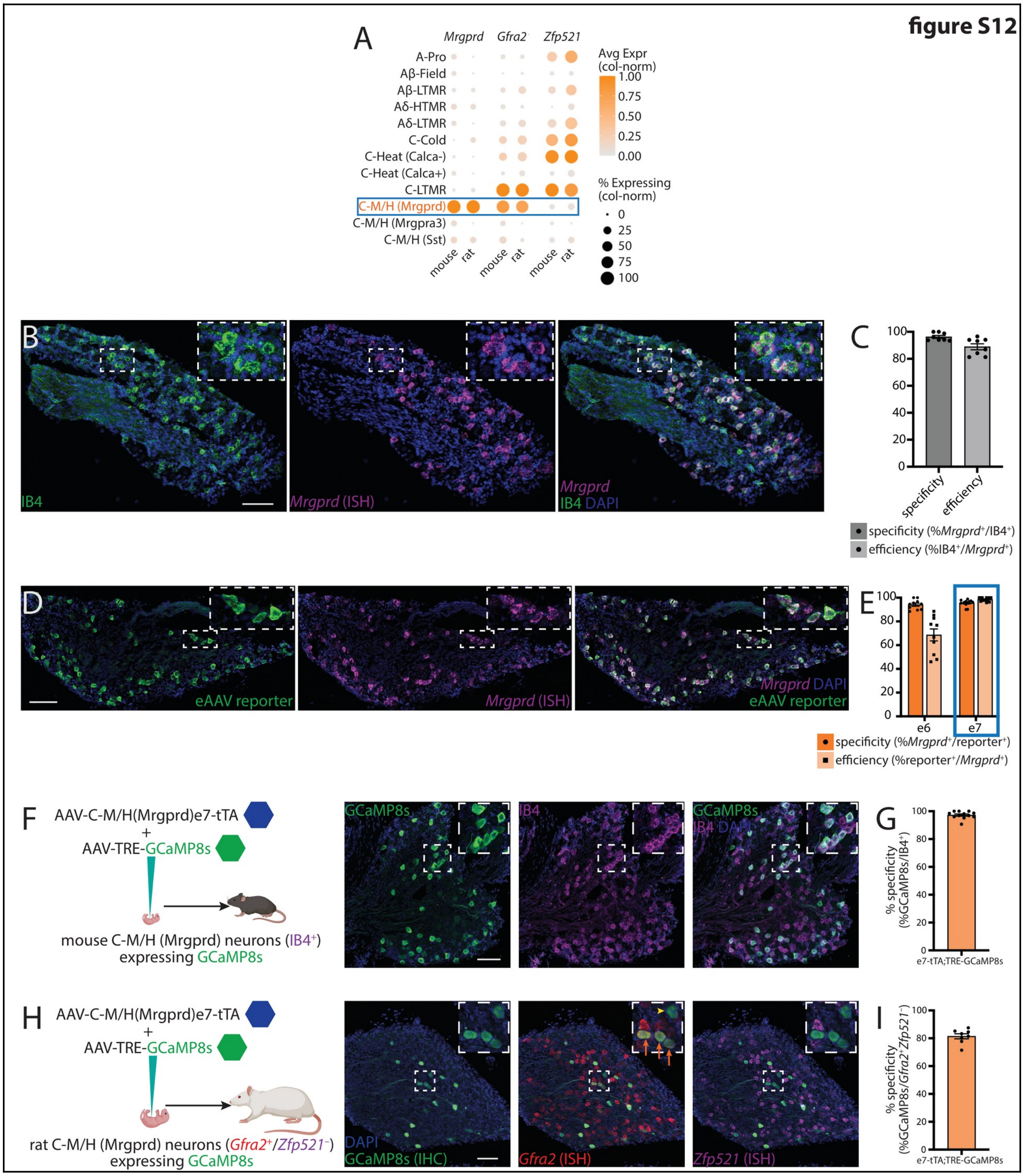

Fig. S12. Additional validation of C-M/H (Mrgprd) neuron eAAV targeting in mice and rats.

(**A**) Dot plot showing expression of *Mrgprd*, *Gfra2*, and *Zfp521* in mouse and rat DRG neuron orthotypes. C-M/H (Mrgprd) neurons are the only DRG neuron orthotype that expresses high *Gfra2* but no appreciable *Zfp521*.

(**B** and **C**) Histology showing that IB4-binding is a property of C-M/H (Mrgprd) neurons in mice, marked by *Mrgprd in situ* hybridization signal. Fluorescence images (B). Quantification of specificity and efficiency (C); mean ± SEM.

bioRxiv preprint doi: https://doi.org/10.64898/2026.09.19.752904; this version posted September 21, 2026. The copyright holder for this preprint (which was not certified by peer review) is the author/funder, who has granted bioRxiv a license to display the preprint in perpetuity. It is made available under a CC-BY 4.0 International license.

(**D** and **E**) Joint *in situ* hybridization-immunohistochemical validation of eAAV payload reporter expression in mouse C-M/H (Mrgprd) neurons (*Mrgprd*$^{+}$). Fluorescence images (D). Quantification of specificity and efficiency (E); mean ± SEM. Blue box indicates eAAV selected for use in further experiments.

(**F** to **I**) Schematics illustrating viral strategy for high GCaMP8s expression in mouse (F) and rat (H) C-M/H (Mrgprd) neurons. Fluorescence images displaying GCaMP8s expression in mouse (F, IB4^{+}) and rat (H, *Gfra2*$^{+}$/*Zfp521*$^{-}$) C-M/H (Mrgprd) neurons. Quantification of specificity of GCaMP8s expression in mouse (G) and rat (I) C-M/H (Mrgprd) neurons; mean ± SEM.

Scale bars, 100 µm.

bioRxiv preprint doi: https://doi.org/10.64898/2026.09.19.752904; this version posted September 21, 2026. The copyright holder for this preprint (which was not certified by peer review) is the author/funder, who has granted bioRxiv a license to display the preprint in perpetuity. It is made available under a CC-BY 4.0 International license.

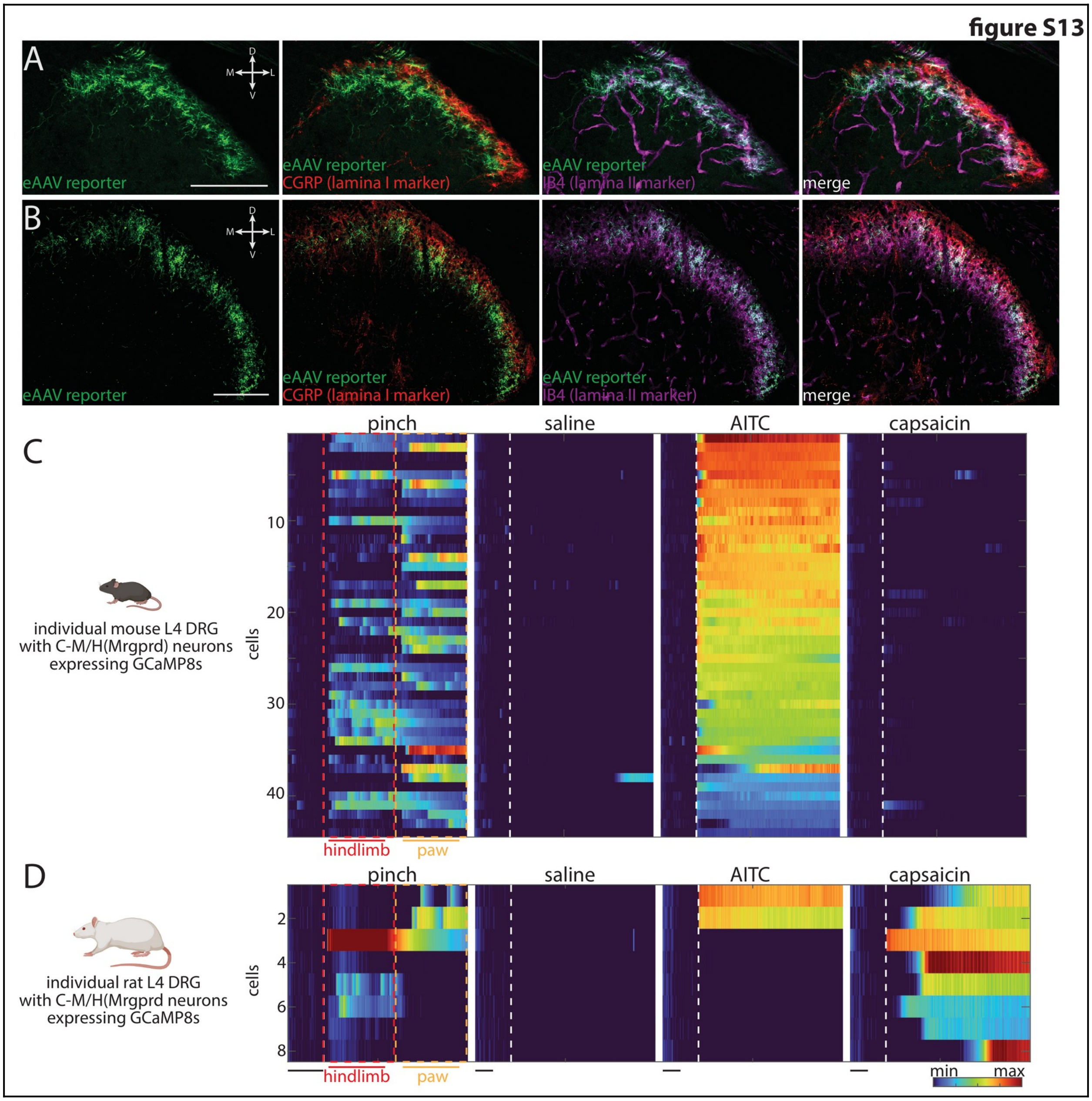

Fig. S13. Additional characterization of eAAV-mediated C-M/H (Mrgprd) neuron anatomy and physiology in mice and rats.

(**A** and **B**) eAAV-mediated C-M/H (Mrgprd) neuron labeling in mice (A) and rats (B) reveals central terminals targeting spinal cord dorsal horn lamina II marked by overlap with IB4-binding signal. Scale bars, 100 µm. See also fig. S6G.

(**C** and **D**) Example Ca^{2+} imaging results from single mouse (C) and rat (D) DRGs displaying responses to pinch, vehicle control (saline), and chemical stimuli. Dotted white lines, stimulus onset. Colored bar, fluorescence (ΔF/F). Black bars, 10 seconds.

bioRxiv preprint doi: https://doi.org/10.64898/2026.09.19.752904; this version posted September 21, 2026. The copyright holder for this preprint (which was not certified by peer review) is the author/funder, who has granted bioRxiv a license to display the preprint in perpetuity. It is made available under a CC-BY 4.0 International license.

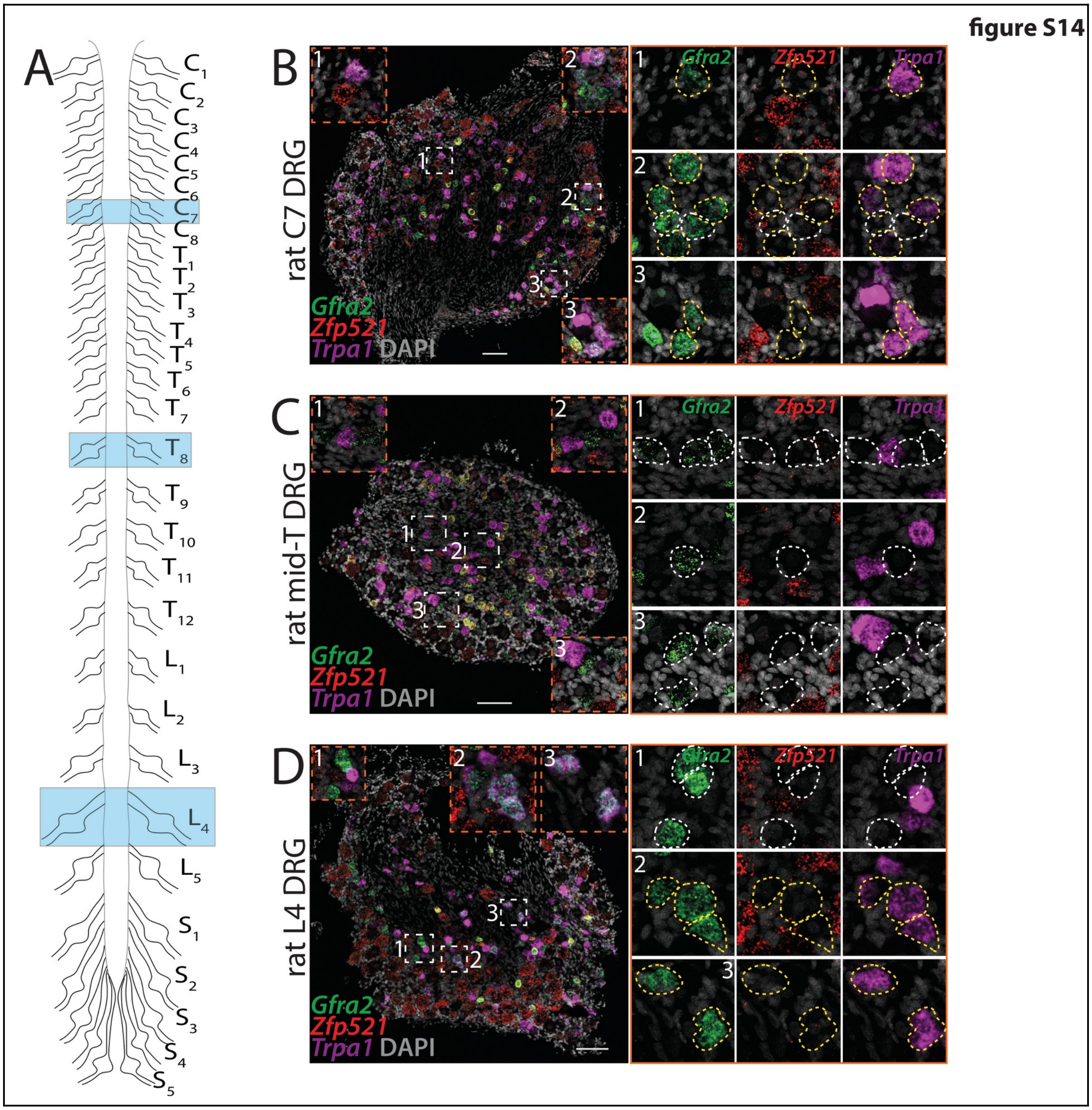

Fig. S14. Histological identification of rat C-M/H (Mrgprd) neuron paratypes across axial levels.

(**A**) Schematic illustrating DRGs taken of different axial levels. Note that cervical 7 (C7) and lumbar 4 (L4) axial level DRGs innervate distal limbs while mid-thoracic (mid-T, e.g., T8) axial level DRGs do not (see also Fig. 6C).

(**B** to **D**) *In situ* hybridization images showing rat C-M/H (Mrgprd)$_1$ (*Trpa1*$^-$) and C-M/H (Mrgprd)$_2$ (*Trpa1*$^+$) neuron paratypes across C7, mid-T, and L4 DRGs. C-M/H (Mrgprd) neurons are *Gfra2*$^+$/*Zfp521*$^-$ (fig. S12A) and are indicated in the insets (right) by dotted white (*Trpa1*$^-$) and yellow (*Trpa1*$^+$) outlines. See quantification in Fig. 6, D to F. Scale bars, 100 µm.

bioRxiv preprint doi: https://doi.org/10.64898/2026.09.19.752904; this version posted September 21, 2026. The copyright holder for this preprint (which was not certified by peer review) is the author/funder, who has granted bioRxiv a license to display the preprint in perpetuity. It is made available under a CC-BY 4.0 International license.

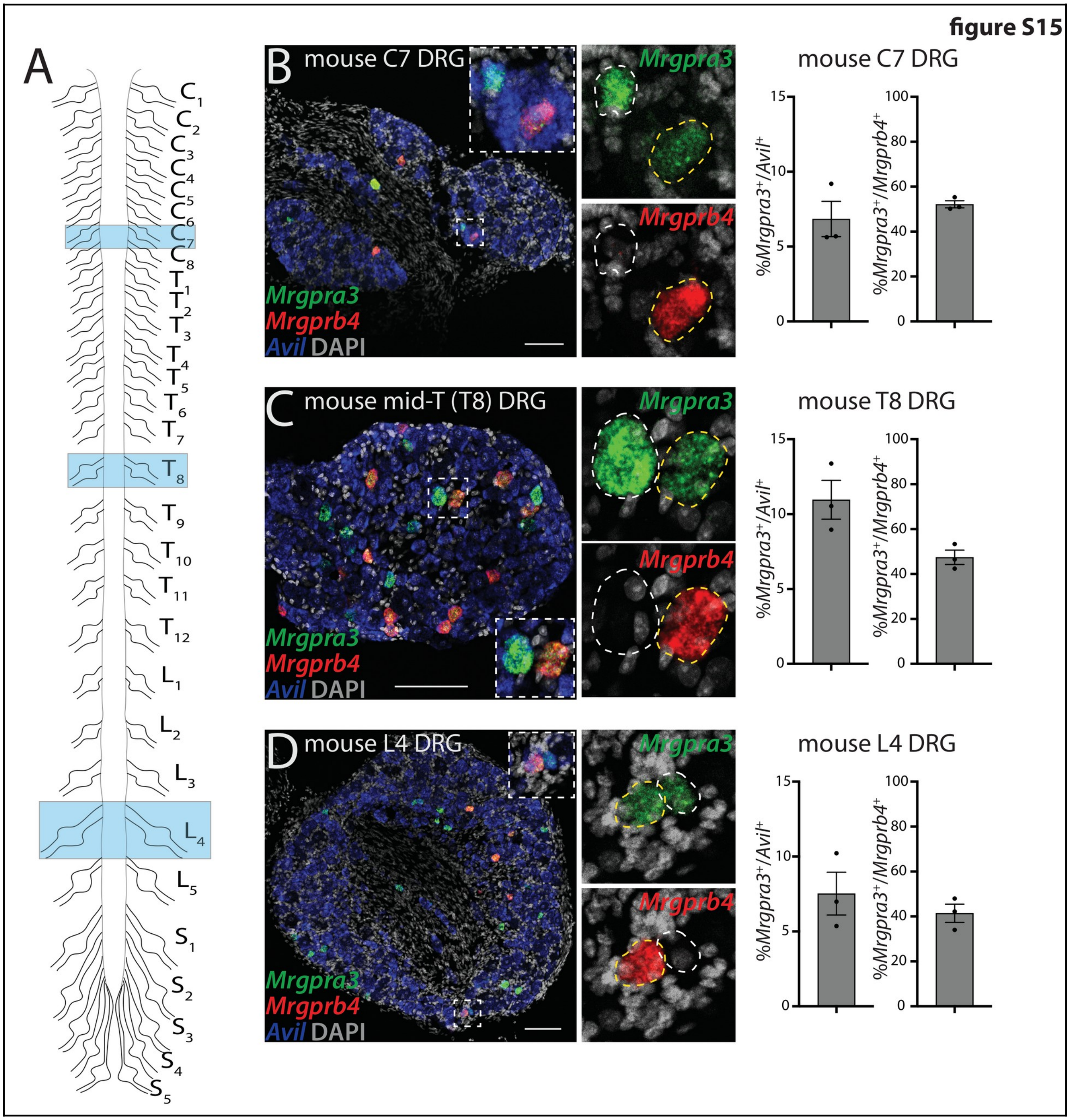

Fig. S15. Histological identification of mouse C-M/H (Mrgpra3) neuron paratypes across axial levels.

(**A**) Schematic illustrating DRGs taken of different axial levels. Note that cervical 7 (C7) and lumbar 4 (L4) axial level DRGs innervate distal limbs while mid-thoracic (mid-T, e.g., T8) axial level DRGs do not (see also Fig. 6C). (**B** to **D**) *In situ* hybridization images (left) and percentages (right) of mouse C-M/H $(Mrgpra3)_1$ (only *Mrgpra3*$^+$) and C-M/H $(Mrgpra3)_2$ (*Mrgpra3*$^+$*Mrgprb4*$^+$) neuron paratypes across C7, mid-T (T8), and L4 DRGs; mean ± SEM. Scale bars, 100 µm.

bioRxiv preprint doi: https://doi.org/10.64898/2026.09.19.752904; this version posted September 21, 2026. The copyright holder for this preprint (which was not certified by peer review) is the author/funder, who has granted bioRxiv a license to display the preprint in perpetuity. It is made available under a CC-BY 4.0 International license.

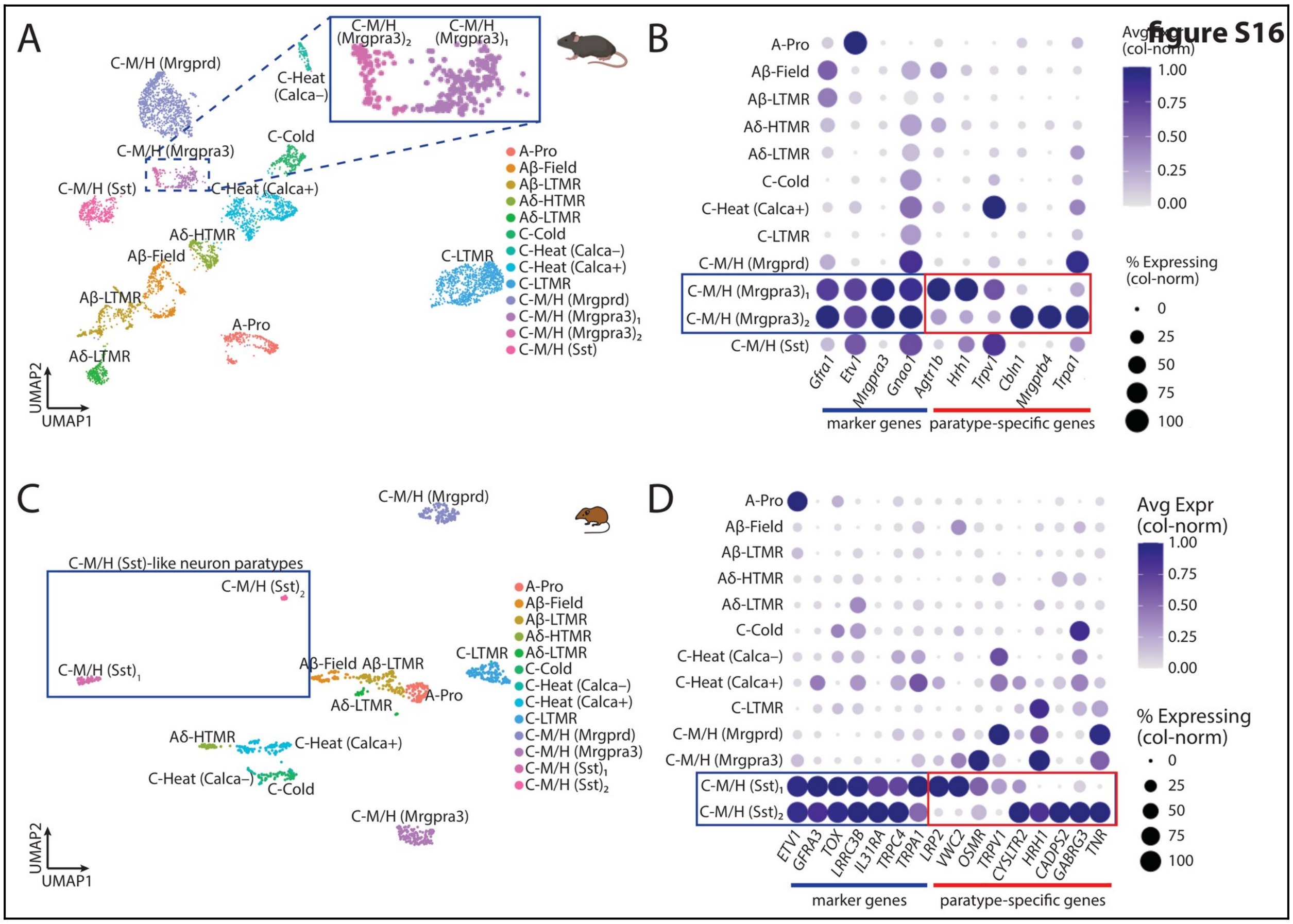

Fig. S16. Bioinformatic identification and characterization of mouse C-M/H (Mrgpra3) and Etruscan shrew C-M/H (Sst) neuron paratypes.

(**A**) UMAP embedding of snRNA-seq data from an individual mouse sample replicate. Colors indicate somatosensory neuron orthotype identities. Inset shows splitting of C-M/H (Mrgpra3) neuron orthotype into two putative paratypes.

(**B**) Dot plot showing expression of common, orthotype-defining marker genes (blue line/box) and paratype-defining positive marker genes (red line/box) of mouse C-M/H (Mrgpra3) neuron paratypes.

(**C**) UMAP embedding of snRNA-seq data from an individual shrew sample replicate. Colors indicate somatosensory neuron orthotype identities. Box shows splitting of C-M/H (Sst) neuron orthotype into two putative paratypes.

(**D**) Dot plot showing expression of common, orthotype-defining marker genes (blue line/box) as well as paratype-defining positive marker genes (red line/box) of shrew C-M/H (Sst) neuron paratypes.
